# Deciphering the Metabolomics-Based Intervention of Yanghe Decoction on Hashimoto's Thyroiditis

**DOI:** 10.1155/2022/6215573

**Published:** 2022-07-15

**Authors:** Xing Zhang, Dexuan Chen, Kun Xu, Zhaoqun Ma

**Affiliations:** Surgery of Chinese Medicine, Nanjing University of Chinese Medicine Affiliated Yancheng Chinese Medicine Hospital, No. 56 Renmin Road, Yancheng 224000, China

## Abstract

**Background:**

Yanghe decoction is a famous formula consisting of *Rehmannia*, deer horn gum, cinnamon, rue, *Ephedra*, ginger charcoal, and licorice. However, few studies have explored the role of the potential mechanism of Yanghe decoction in the treatment of Hashimoto's thyroiditis by metabolomics.

**Methods:**

Nine mice were randomly divided into three groups: control group (group C), model group (group M), and drug administration group (group T), with three mice in each group. Mice in groups M and T were established as models of Hashimoto's thyroiditis, and group T was treated with Yanghe decoction. The metabolome of plasma samples from each group of mice was determined using mass spectrometry coupled with high-performance liquid and gas phases, and nuclear magnetic resonance. Based on the three assays, principal component analysis was performed on all samples, as well as orthogonal partial least squares-discriminant analysis and differential metabolite molecules for groups M and T. Subsequently, pathway enrichment analysis was performed, and the intersection was taken for the differential metabolites screened in the M and T groups. The levels of inflammatory factors IL-35 and IL-6 within the serum of each group of mice were detected.

**Results:**

The difference analysis showed that a total of 38 differential metabolites were screened based on mass spectrometry coupled with the high-performance liquid phase, 120 differential metabolites were screened based on mass spectrometry coupled with gas phase, and a total of *α*-glucose and *β*-glucose were the differential metabolites analyzed based on NMR test results. The pathways enriched by the differential metabolites in the M and T groups were intersected, and a total of 5 common pathways were obtained (amino acid tRNA biosynthesis, D-glutamine and D-glutamate metabolism, tryptophan metabolism, nitrogen metabolism, and arginine and proline metabolism). The results also showed a significant decrease in the serum inflammatory factor IL-35 and a significant increase in IL-6 in mice from group M compared with group C, while a significant increase in the serum inflammatory factor IL-35 and a significant decrease in IL-6 in mice from group T compared with group M.

**Conclusion:**

Our study reveals the metabolites as well as a metabolic network that can be altered by Yanghe decoction treatment of Hashimoto's thyroiditis and shows that Yanghe decoction can effectively reduce the level of inflammatory factors in Hashimoto's thyroid.

## 1. Introduction

Hashimoto's thyroiditis, also known as chronic lymphocytic or autoimmune thyroiditis, is an autoimmune thyroid disease that causes the immune system to attack and destroy the thyroid gland [1]. It is characterized by an enlarged thyroid gland, parenchymal lymphocytic infiltration, and the presence of thyroid antigen-specific antibodies [2]. Hashimoto's thyroiditis causes chronic inflammation of the thyroid tissue and may result in hypothyroidism in 20–30% of patients [3]. The incidence is approximately 3 to 6 cases per 10,000 people per year, with a prevalence of at least 2% in women. The glands involved in thyroiditis often lose the ability to store iodine, produce and secrete rod proteins that circulate in the plasma, and fail to make hormones efficiently [4,5]. At present, methylprednisolone is often used in clinical practice for Hashimoto's thyroiditis treatment, which could inhibit the synthesis of the thyroid gland and alleviate the patient's clinical symptoms. However, research has shown that the use of methimazole alone in patients with Hashimoto's thyroiditis for long term often have poor prognosis.

Traditional Chinese medicine is remarkably effective in the adjuvant treatment of the disease, especially in improving clinical symptoms, prolonging patient survival, and modulating immune function. As an effective method for preventing and treating diseases, TCM has been increasingly used worldwide in the past decades [6]. Yanghe decoction is a famous formula consisting of *Rehmannia*, deer horn gum, cinnamon, rue, *Ephedra*, ginger charcoal, and licorice [7]. It has the effect of warming yang and nourishing blood and dispersing cold and moving stagnation. For centuries, Yang He Tang has been proven to be used to treat a variety of noninfectious inflammatory conditions [8].

Yanghe decoction can be combined with modern therapies, which have a combination of “multicomponent, multitarget, and multipathway” regulatory mechanisms and have fewer toxic side effects [9]. In one study, Yang He Tang was shown to have a high mean cure rate (defined as complete regression or significant improvement of lumps and pain for at least two months) in patients with chronic breast fibrosis and palpable lumps [10]. Fewer studies have clinically investigated the effect of Yanghe decoction in the treatment of Hashimoto's thyroiditis. Therefore, the present study was conducted to investigate the potential mechanism of Yanghe decoction in the treatment of Hashimoto's thyroiditis through a metabolomic approach.

## 2. Material and Methods

### 2.1. Animal Grouping and Model Construction

Nine NOD mice (4 weeks old) were purchased from the Nanjing Biomedical Research Institute of Nanjing University. NOD mice were randomly divided into three groups: control group (group C), model group (group M), and drug administration group (group T), with three mice in each group. The mice in the model group and the drug administration group were treated with porcine thyroglobulin and high iodine water to establish the mouse model of autoimmune thyroiditis. After successful modeling, Yanghe decoction formula granule (Xuyang Pharmaceutical Co., LTD., China) was prepared into 0.5 g/mL liquid medicine; the drug-administered group was given 0.5 g/ml Yanghe decoction formula 1 ml/100 g by gavage for 1 h, and the model group and normal group were given the same amount of saline by gavage once a day for 10 weeks. The serum and plasma samples were collected and stored at −20°C for serum and −80°C for plasma. The study protocols were approved by the Institutional Animal Care and Use Committee of Nanjing University of Chinese Medicine Affiliated Yancheng.

### 2.2. Enzyme-Linked Immunosorbent Assay

The serum collected was assayed for IL-35 (interleukin-35) and IL-6 (interleukin-6) inflammatory factors using enzyme-linked immunosorbent assay (ELISA) kits. The experiment was performed according to the instructions.

### 2.3. Mass Spectrometry Coupled with Ultra-High Performance Liquid-Phase Detection

Metabolite extraction was performed in strict accordance with the operating instructions, followed by onboard detection.

*On-Board Detection*. The target compounds were chromatographed on a Waters ACQUITY UPLCBEH Amide (2.1 mm × 100 mm, 1.7 *μ*m) column using a Vanquish (Thermo Fisher Scientific) ultra-performance liquid chromatography. The A phase of the liquid chromatography was aqueous containing 25 mmol/L ammonium acetate and 25 mmol/L ammonia, and the B phase was acetonitrile. The sample tray temperature is 4°C, and injection volume is 2 *μ*L.

The Thermo Q Exactive HFX mass spectrometer was capable of primary and secondary mass spectrometry data acquisition under the control of the control software Xcalibur (Thermo). Detailed parameters were as follows: sheath gas flow rate, 30 Arb; Aux gas flow rate, 25 Arb; capillary temperature: 350°C; full ms resolution, 60000; MS/MS resolution, 7500; collision energy, 10/30/60 in NCE mode; and spray voltage: 3.6 kV (positive) or −3.2 kV (negative).

*Data Processing*. The raw data were converted to mzXML format by ProteoWizard software, and then the peak identification, peak extraction, peak alignment, and integration were performed using the R package (kernel XCMS) written by ourselves and then matched with the BiotreeDB (V2.1) self-built secondary mass spectrometry database for substance annotation.

### 2.4. Mass Spectrometry Coupled with Gas Chromatography Detection

Metabolite extraction was performed strictly according to the operating instructions, and then all samples were analyzed by gas chromatography and time-of-flight mass spectrometry.

*On-Board Detection*. GC-TOF-MS analysis was performed using an Agilent 7890 gas chromatograph and a time-of-flight mass spectrometer. The system used a DB-5MS capillary column. 1 *μ*L aliquots were injected in a nonsplit mode. Helium was used as the carrier gas with a front inlet purge flow rate of 3 mL min^−1^ and a gas flow rate through the column of 1 ml min^−1^. The initial temperature was held at 50°C for 1 min, then increased to 310°C at a rate of 20°C, and then held at 310°C for 6 min. The injection, transmission line, and ion source temperatures were 280, 280, and 250°C, respectively. The energy in electron collision mode was −70 eV. After a solvent delay of 4.83 min, mass spectral data were acquired in full-scan mode in the m/z range of 50–500 at a rate of 12.5 spectra per second.

*Data Processing*. The mass spectral data were analyzed for peak extraction, baseline correction, deconvolution, peak integration, and peak alignment using ChromaTOF software (V 4.3x, LECO). For the substance characterization work, the LECO-Fiehn Rtx5 database was used, including mass spectrometry matching and retention time index matching.

### 2.5. Nuclear Magnetic Resonance Detection

The experimental testing equipment was Varian Inova 600M Agilent.

*Description of the Spectral Processing*. The integration interval of the serum NMR spectra was 0.5–8.5 ppm with an integration spacing of 0.002 ppm, and a section of 4.60–4.80 ppm containing the residual water peak and the urea peak at 5.20–5.25 ppm and the region of its influence was removed. Among them, the demonstration of mouse serum spectra for groups C, M, and T were chosen: 1, 9, and 17, respectively.

### 2.6. Principal Component Analysis (PCA)

Data were formatted for logarithmic (LOG) transformation plus centralization (CTR) using SIMCA software (V16.0.2; Sartorius Stedim Data Analytics AB, Umea, Sweden), followed by automated modeling analysis.

### 2.7. Orthogonal Partial Least Squares-Discriminant Analysis (OPLS-DA)

The data were UV-formatted using SIMCA software (V14.1; MKS Data Analytics Solutions, Umea, Sweden), and OPLS-DA modeling analysis was first performed on the first principal component, and the quality of the model was tested with 7-fold cross-validation (7-fold cross-validation). Then, R2Y (interpretability of the model for the categorical variable Y) and Q2 (predictability of the model) were used to judge the validity of the model; finally, the validity of the model was further tested by a permutation test, which randomly changed the order of the categorical variable Y several times to obtain different random Q2 values.

### 2.8. Pathway Enrichment Analysis

The metabolic pathways of differentially expressed metabolites were analyzed by searching the relevant metabolic pathways of differentially expressed metabolites using authoritative metabolite databases such as Kyoto Encyclopedia of Genes and Genomes (KEGG) and PubChem.

## 3. Results

### 3.1. Principal Component Analysis

**Figure 1:**
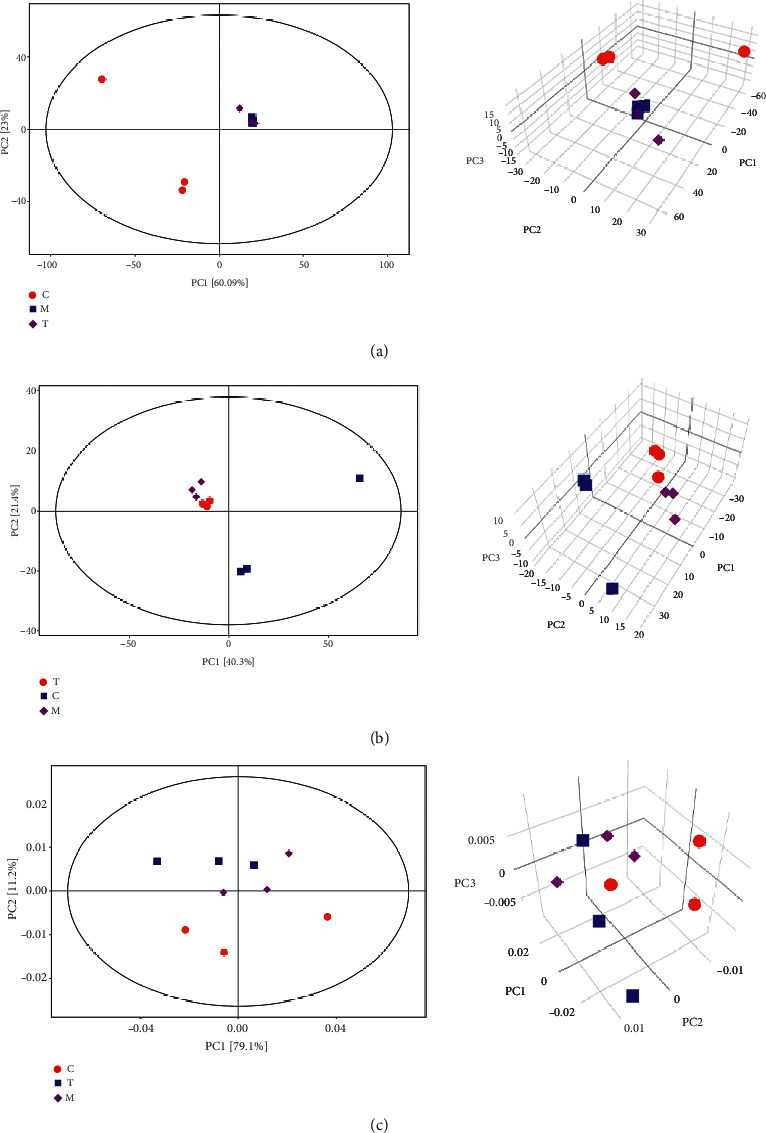
Principal component analysis. (a) Scatter plot and 3D plot of PCA scores for all samples detected by mass spectrometry coupled with the high-performance liquid phase. (b) Scatter plot and 3D plot of PCA scores for all samples detected by mass spectrometry coupled with the gas phase. (c) Scatter plot and 3D plot of PCA scores of all samples detected by NMR.

First, we performed PCA on the results of the three assays to observe the overall metabolic levels and species differences between the groups. The results of principal component analysis (positive ions) of the mass spectrometry coupled with gas and high-performance liquid phases showed that the samples of groups M and T were more closely distributed and both groups were distant from the samples of group C. The results of the NMR principal component analysis showed that the samples of groups M and T were distributed more into and partitioned from the samples of group C. This can all indicate that the more similar the type and content of metabolites in groups M and T, the more different the overall metabolic levels in groups M and T from group C. The samples were all in the 95% confidence interval. See Figure 1.

### 3.2. Orthogonal Partial Least Squares-Discriminant Analysis (OPLS-DA) and Differential Metabolite Analysis

**Figure 2:**
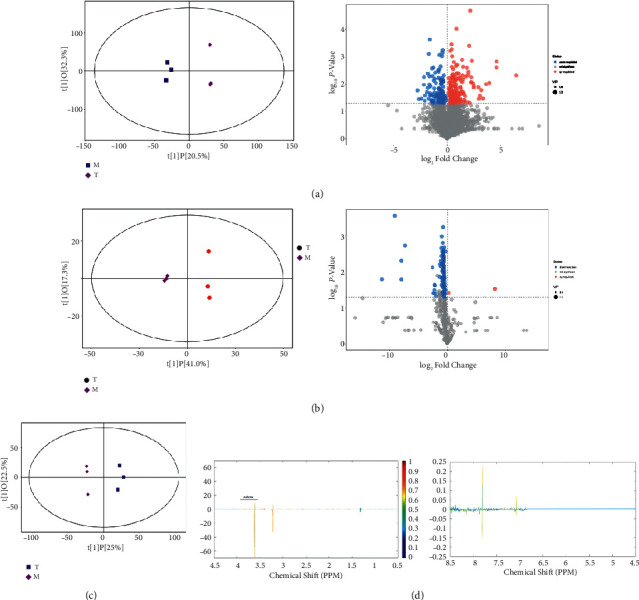
OPLS-DA model score scatter plot and differential metabolite analysis. (a) Scatter plot of OPLS-DA model score and volcano plot of differential metabolites for group M versus group T detected by mass spectrometry coupled with the high-performance liquid phase. (b) Scatter plot of OPLS-DA model score and volcano plot of differential metabolites for group M versus group T detected by mass spectrometry coupled with gas phase. The size of scattering in the volcano plot represents the VIP value of OPLS-DA model, the larger the scatter the larger the VIP value. Significantly upregulated metabolites are shown in red, significantly down-regulated metabolites are shown in blue, and nonsignificantly different metabolites are shown in gray. (c) Scatter plot of OPLS-DA model scores for group M versus group T detected by NMR. (d) OPLS-DA loadings plot for group M versus group (T). The horizontal coordinates in the plot indicate the magnitude of chemical shifts, from right to left, with increasing values of chemical shifts; the vertical coordinates indicate the magnitude of loadings after back conversion, where the shades of color indicate the magnitude of correlation coefficients, red indicates a positive correlation, and blue indicates negative correlation. The marked substances in the figure are the differential metabolites that passed the critical value test of the correlation coefficient.

We then modeled OPLS-DA for the M and T groups and screened for differential metabolites. The scatter plots of OPLS-DA model scores for the three assays showed that the horizontal distance between samples was farther for group M versus group T, which could indicate the greater the difference between the two groups, and the samples were very clearly differentiated. The samples were all in the 95% confidence interval. The results of the differential metabolite analysis showed that a total of 38 differential metabolites were screened for the mass spectrometry coupled with high-performance liquid phase (Table 1); a total of 120 differential metabolites were screened for the mass spectrometry coupled with gas phase (Table 2), and the conditions for this metabolite screening were VIP value (the projected importance of the variable obtained from the OPLS-DA model for the comparison of the substance in this group) > 1 and *P* < 0.05. NMR analysis showed that *α*-glucose and *β*-glucose were significant differentiating metabolites (see Figure 2).

### 3.3. Enrichment Analysis of Metabolic Pathways of Differential Metabolites

**Figure 3:**
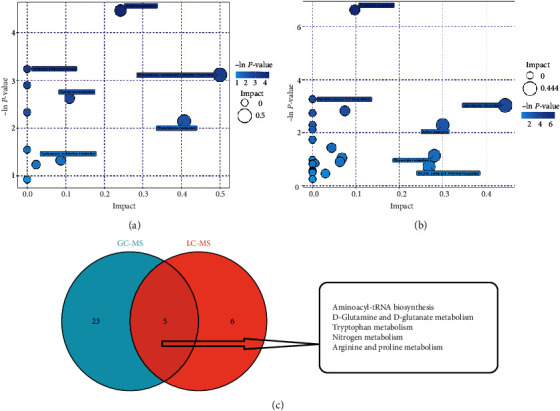
Metabolic pathway enrichment analysis of differential metabolites. (a) Pathway analysis of group M versus group T based on mass spectrometry combined with high-performance liquid chromatography. (b) Pathway analysis of group M versus group T based on mass spectrometry combined with gas chromatography. Each bubble represents a metabolic pathway; the bubble size represents the influence factor, the larger the size, the larger the influence factor; the vertical coordinate of the bubble and the color of the bubble indicate the *P* value of the enrichment analysis; the darker the color, the smaller the *P* value, the more significant the enrichment.

We further analyzed the effect of differential metabolites on their pathways. The analysis based on the mass spectrometry combined with high-performance liquid phase detection results showed that the differential metabolites in the M and T groups were enriched to 11 pathways (Table 3), among which the pathways with higher enrichment were histidine metabolism, amino acid tRNA biosynthesis, phenylalanine, tyrosine and tryptophan biosynthesis, tryptophan metabolism, phenylalanine metabolism, cysteine, and methionine metabolism. The analysis based on the results of mass spectrometry combined with gas-phase detection showed that the differential metabolites of the M and T groups were enriched to 28 pathways (Table 4), among which the pathways with higher enrichment were pyrimidine metabolism, biosynthesis of pantothenic acid and CoA, metabolism of *β*-alanine, sulfur metabolism, glycerolipid metabolism, and metabolism of glycine, serine, and threonine. Subsequently, we took the intersection of the pathways enriched by the differential metabolites in the M and T groups for both high-performance liquid- and gas-phase methods coupled to mass spectrometry and obtained a total of five common pathways (biosynthesis of amino acid tRNA, metabolism of D-glutamine and D-glutamate, metabolism of tryptophan, metabolism of nitrogen, and metabolism of arginine and proline). See Figure 3

### 3.4. Comparison of Serum Inflammatory Factor Levels in Three Groups of Mice

**Figure 4:**
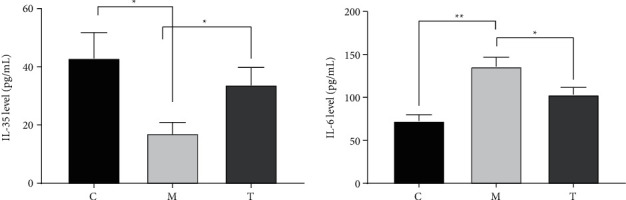
Comparison of serum levels of inflammatory factors IL-35 and IL-6 in three groups of mice.

Finally, we observed the serum inflammatory factor levels in the three groups of mice to determine the effect of Yanghe decoction on inflammatory factors in mice with Hashimoto's thyroiditis. The results showed that the serum inflammatory factor IL-35 was significantly lower and IL-6 was significantly higher in mice of group M than group C, while the serum inflammatory factor IL-35 was significantly higher and IL-6 was significantly lower in mice of group T than group M. The results showed that the serum inflammatory factor IL-35 was significantly lower and IL-6 was significantly lower in mice of group T than group C. See Figure 4.

## 4. Discussion

Metabolism plays a central role as a signaling molecule, immunomodulator, endogenous toxin, and environmental sensor in all areas of biology, from ecology to bioengineering to cancer. Each of these fields is now increasingly being studied from a metabolic perspective. And these studies are valuable from a big picture perspective [11]. The metabolome is a collection of small-molecule chemical entities involved in metabolism and has traditionally been studied to identify biomarkers for the diagnosis and prediction of disease. Nowadays, the value of metabolomic analysis has been redefined from a simple biomarker identification tool to a technique for discovering active drivers of biological processes [12]. Metabolomics is the high-throughput characterization of metabolites from cells, organs, tissues, or biofluids using advanced analytical chemistry techniques [13]. NMR and mass spectrometry are commonly used in metabolomics; NMR is highly reproducible and quantitative, has a simple sample preparation protocol, and is capable of measuring analytes in a variety of solvent conditions, but it has low sensitivity. In contrast, the high sensitivity and low detection limits of mass spectrometry enable the detection of subtle metabolic changes that are not visible with NMR [14]. In this experiment, a total of 38 differential metabolites were screened based on mass spectrometry coupled with the high-performance liquid phase, 120 differential metabolites were screened based on mass spectrometry coupled with gas phase, and a total of *α*-glucose and *β*-glucose were analyzed based on NMR test results.

Measuring metabolite concentrations by metabolomics only tells half the story. Equally important is to understand pathway activity, which can be quantified as the flow of material per unit time, i.e., metabolic flux. In this experiment, we took intersections of pathways enriched by differential metabolites in the M and T groups for both mass spectrometry coupled with high-performance liquid and gas phases, yielding a total of five common pathways, namely, amino acid tRNA biosynthesis, D-glutamine and D-glutamate metabolism, tryptophan metabolism, nitrogen metabolism, and arginine and proline metabolism.

Amino tRNA is a substrate for translation and plays a key role in determining how the genetic code is interpreted into amino acids. The function of aminyl-tRNA synthesis is to precisely match amino acids to tRNAs containing the corresponding anticodons [15]. Aminyl-tRNA synthetase is essential for the physical interpretation of the genetic code [16], and in addition to its function in protein synthesis, it is involved in various cellular processes such as immune and inflammatory responses, angiogenesis, and apoptosis [17,18]. Just one study showed that the pentose phosphate pathway, amyl-tRNA biosynthesis, and pyrimidine metabolism are the main pathways altered in hypothyroidism [19]. Glutamate is a key excitatory neurotransmitter responsible for maintaining cognitive function and neuronal plasticity [20], while metabolites associated with glutamate metabolism, 2-ketoglutarate, L-aspartate, and fumarate are associated with the gut microbiota, and their alterations may affect human health [21]. Some studies have indicated that glycerophospholipid, glutamine, and glutamate metabolism, and related metabolites are potential key targets for common molecular mechanisms linking HIV to NCDs through inflammation and oxidative stress [22]. Tryptophan is an essential aromatic amino acid consisting of a *β*-carbon attached to the 3-position of the indole group. Although tryptophan is the least abundant amino acid in proteins and cells, it is a biosynthetic precursor for a large number of microbial and host metabolites [23,24]. Tryptophan metabolism in the intestine is the direct conversion of tryptophan by intestinal microorganisms into several molecules, such as indoles and their derivatives. And many of these indole derivatives, in turn, are ligands for aryl hydrocarbon receptors [25]. Aryl hydrocarbon receptor signaling is thought to be a key component of the immune response at the barrier site and thus can maintain intestinal homeostasis by acting on epithelial renewal, barrier integrity, and many immune cell types [26]. Arginine is a nonessential or semiessential amino acid that plays an important role in a variety of biological functions including cell proliferation, survival, and protein synthesis. It is also a precursor for the production of nitric oxide, polyamines, proline, creatinine, and glutamate. As a multifunctional amino acid, arginine plays an important role in physical health by being involved in tissue damage and chronic metabolic diseases [27]. Arginine has also been associated with endothelial function, inflammation, and airway hyperresponsiveness [28]. Just one study indicated that arginine and proline metabolic pathways are related to asthma pathogenesis [29]. The above combined with the results of the present experiment could suggest that Yanghe decoction may maintain homeostasis by altering the relevant metabolic pathways and thus improving the disease. In addition, the above pathways have shown relevance to inflammation as well as immune cells. For this reason, we also investigated the effect of Yanghe decoction.

Inflammation is a comprehensive physiological response to tissue damage, which is caused by physical injury, infection, exposure to toxins, or other types of trauma [30]. There is growing evidence that inflammation is a major factor in the progression of many diseases, including autoimmune thyroiditis [31]. The results of the present study showed that serum inflammatory factor IL-35 was significantly lower and IL-6 was significantly higher in mice in the model group compared with the normal control group, whereas serum inflammatory factor IL-35 was significantly higher and IL-6 was significantly lower in mice treated based on Yanghe decoction compared with the model group. This may be due to the effect of one or a combination of herbs in the composition of Yanghe decoction. Deer antler tablets have antifatigue, anti-inflammatory, and analgesic effects, while deer antler peptides are the main active ingredients obtained by isolation from deer antler tablets [32]. It has been shown that in osteoblasts, antler peptides block TNF-*α*-mediated inhibition of osteoblastogenesis and inhibit osteoclastogenesis through the nuclear factor-*κ*B (NF-*κ*B)/p65 pathway. In addition, deer antler peptides reduce levels of interleukin 1*β* and interleukin 6, as well as oxidative responses induced by increased catalase activity and reduced malondialdehyde levels [33]. Previous studies have also indicated that Yanghe decoction may improve the symptoms of Hashimoto's thyroiditis and reduce inflammation [34]. This could suggest that Yanghe decoction can effectively reduce the inflammatory response in Hashimoto's thyroiditis, but whether the specific mechanism is related to metabolic pathways remains to be explored.

## 5. Conclusion

In summary, we detected multiple metabolites that can be altered by Yanghe decoction by NMR and mass spectrometry coupled with gas or liquid chromatography, and most of them were related to aminyl-tRNA biosynthesis, D-glutamine and D-glutamine metabolism, tryptophan metabolism, nitrogen metabolism, and arginine and proline metabolic pathways. In addition, Yanghe decoction can effectively reduce serum inflammatory factor levels in mice with Hashimoto's thyroiditis.

Although we identified the metabolites that can be altered by Yanghe decoction and the pathways that are highly affected, there are still shortcomings in this study. We only observed the metabolomic profile of plasma, and further studies should evaluate serum, urine, cerebrospinal fluid, and brain samples to accurately reflect the pathological changes in Hashimoto's thyroiditis and the therapeutic mechanisms of Yanghe decoction.

## Figures and Tables

**Table 1:** Differential metabolites detected by mass spectrometry and high-performance liquid chromatography.

ID	MS2 name	MS2 score	rt	mz	SuperClass	Type	MEAN T	MEAN M	VIP	*P*-value	Q-value	FOLDCHANGE	LOG_FOLDCHANGE
22	D-Glutamine	0.999291	389.5485	147.0759376	Organic acids and derivatives	Forward	3.746183952	3.446653347	1.931664281	0.014765644	1	1.086904767	0.120225539
47	Trigonelline	0.995484	301.0875	138.0543287	Alkaloids and derivatives	Forward	1.024957601	0.838952133	2.022165048	0.046051103	1	1.221711658	0.288903828
54	N-Acetylhistamine	0.994856	147.787	154.0968957	Organic acids and derivatives	Forward	0.073990814	0.110698454	1.829180635	0.025585478	1	0.668399709	−0.58121699
56	Kanzonol K	0.994656	35.0689	437.1927582	Phenylpropanoids and polyketides	Forward	0.051954891	0.031048015	2.101281275	0.001974289	1	1.673372393	0.742758539
69	L-Methionine	0.991602	301.415	150.0577251	Organic acids and derivatives	Forward	0.616919645	0.468069797	1.939910681	0.019678144	1	1.318007803	0.398358912
99	Ecgonine	0.981134	350.546	186.1120848	Alkaloids and derivatives	Forward	0.027347509	0.030654513	1.926689881	0.031693069	1	0.892120183	−0.164690017
101	L-histidine	0.979726	408.5865	156.0761942	Organic acids and derivatives	Forward	0.49405337	0.223894139	2.08197046	0.008582433	1	2.206638244	1.141850134
107	L-phenylalanine	0.977905	275.944	166.0854692	Organic acids and derivatives	Forward	4.763771466	3.828460241	1.897216209	0.034322892	1	1.244304803	0.315339929
112	L-alanine	0.975801	363.879	90.05507238	Organic acids and derivatives	Forward	0.437920469	0.363410363	1.912131498	0.031994707	1	1.205030219	0.269069326
138	Phytosphingosine	0.965381	57.8408	318.299383	Organic nitrogen compounds	forward	1.743529266	1.126406912	1.948385533	0.021992696	1	1.547868047	0.630282489
141	Pentanenitrile	0.962912	266.604	84.08082795	Organonitrogen compounds	Forward	0.264520137	0.226518588	2.005800964	0.018824486	1	1.167763487	0.223748107
153	Imidazoleacetic acid riboside	0.954489	370.1495	259.0919389	Nucleosides, nucleotides, and analogues	Forward	0.014965502	0.019469476	2.040287972	0.006044593	1	0.768664826	−0.379573442
156	3beta,6beta-Dihydroxynortropane	0.948011	52.1612	144.1012842	Alkaloids and derivatives	Forward	0.118818145	0.086495728	2.050482112	0.004431311	1	1.373688018	0.458054387
158	L-kynurenine	0.944886	280.281	209.0914398	Organic oxygen compounds	Forward	0.10372474	0.082802841	1.987721923	0.013173077	1	1.252671263	0.32500786
160	Arecaidine	0.944798	282.167	142.0858524	Alkaloids and derivatives	Forward	0.080085294	0.053014131	2.016796656	0.022004872	1	1.510640518	0.595160389
161	Carnosine	0.942805	434.3945	227.113435	Organic acids and derivatives	Forward	0.048044936	0.066372136	1.819056239	0.031112771	1	0.723872074	−0.466193334
169	Thelephoric acid	0.935613	407.237	353.0278188	Organoheterocyclic compounds	Forward	0.002605381	0.003775405	1.806140708	0.033212017	1	0.690093214	−0.535136849
180	L-acetylcarnitine	0.92253	320.788	204.1225337	Lipids and lipid-like molecules	Forward	3.698529842	2.839908003	1.877147018	0.024898946	1	1.302341427	0.381107721
204	Alpha-methylstyrene	0.898642	35.2889	119.0850948	Benzenoids	Forward	0.188441473	0.131632093	2.159887496	0.001577729	1	1.431576978	0.517605248
228	Glycerophosphocholine	0.86945	400.206	258.1089005	Lipids and lipid-like molecules	Forward	0.615951963	0.925741511	1.864643532	0.035821933	1	0.665360638	−0.587791573
230	1-(beta-D-ribofuranosyl)-1,4-dihydronicotinamide	0.865109	171.668	257.1128126	Organic oxygen compounds	Forward	0.117325608	0.138566581	2.138035911	0.004789509	1	0.846709269	−0.240061413
238	D-biopterin	0.853104	272.631	238.0928999	Organoheterocyclic compounds	Forward	0.026486036	0.021446249	1.82825674	0.040439888	1	1.234996232	0.30450664
240	N-(3-methylbutyl)acetamide	0.850798	268.9475	130.1221116	Organic acids and derivatives	Forward	0.058209581	0.049743365	2.142098263	0.000620136	1	1.170197912	0.226752549
254	N-acetylornithine	0.829892	374.192	175.1070165	Organic acids and derivatives	Forward	0.056279893	0.047008306	1.830587028	0.035370672	1	1.197232943	0.259703882
259	1-(Hydroxymethyl)-5,5-dimethyl-2,4-imidazolidinedione	0.818992	407.131	159.0757679	Organoheterocyclic compounds	Forward	0.182086983	0.15583556	1.945664927	0.014442075	1	1.168455928	0.224603319
291	Biochanin A	0.769766	39.59165	285.07362	Phenylpropanoids and polyketides	forward	0.031721703	0.041732531	1.920254712	0.018257478	1	0.760119332	−0.395702167
295	Heptadecanoyl carnitine	0.756939	194.733	414.3571643	Lipids and lipid-like molecules	Forward	0.017403164	0.022118651	2.137696573	0.00072227	1	0.786809469	−0.345913775
315	3-[(3-Methylbutyl)nitrosoamino]-2-butanone	0.715231	36.73805	187.1434871	Organooxygen compounds	Forward	0.339965307	0.457120856	2.061300025	0.008977331	1	0.743709903	−0.427188112
320	2-Acetylpyrazine	0.710279	330.908	123.0549956	Organooxygen compounds	Forward	0.013123324	0.008784152	1.970470389	0.02020786	1	1.493977369	0.579158294
328	Sedoheptulose 1-phosphate	0.700854	40.022	291.0477486	Organic oxygen compounds	Forward	0.051097691	0.069345806	2.046508406	0.007069591	1	0.736853377	−0.440550522
360	Glechomafuran	0.646208	224.3045	231.1407859	Lipids and lipid-like molecules	Forward	0.010033971	0.014750196	2.095991059	0.002844627	1	0.680260196	−0.55584142
384	LysoPC(17 : 0)	0.611195	214.164	510.3549507	Lipids and lipid-like molecules	Forward	0.772031639	1.046868823	1.889934534	0.045513658	1	0.73746741	−0.4393488
410	Acetyl-N-formyl-5-methoxykynurenamine	0.577916	24.7427	265.1210007	Organic oxygen compounds	Forward	0.010452578	0.006475994	2.18949614	9.71037E−05	1	1.614049998	0.690685269
430	Cysteinyl-serine	0.560691	25.0757	209.0581161	Organic acids and derivatives	Forward	0.542806005	0.285681699	1.941743744	0.041010554	1	1.900037726	0.926028064
435	L-octanoylcarnitine	0.556023	224.21	288.2159451	Lipids and lipid-like molecules	Forward	0.064410974	0.089677252	2.106970841	0.002284565	1	0.718253205	−0.47743557
442	S-methylmethionine	0.553207	336.697	164.0731609		Forward	0.032573318	0.044869274	1.846945723	0.032907818	1	0.725960445	−0.462037152
471	4-Aminohippuric acid	0.488799	315.061	184.0895643	Benzenoids	Forward	0.009328513	0.023072876	1.911670897	0.010251036	1	0.404306452	−1.306478867
477	PE(16 : 0/18 : 3(9Z,12Z,15Z))	0.471196	164.6285	714.5059277	Lipids and lipid-like molecules	Forward	0.043175276	0.02733873	1.838157839	0.032662642	1	1.579271456	0.659259173
ID	The unique data number of the substance in this qualitative analysis												
MS2 name	The substance name obtained by qualitative matching analysis of secondary mass spectrometry												
MS2 score	The score of the second-level match, with the value [0, 1]												
rt	Chromatographic retention time of the substance												
mz	Material characteristics mass charge ratio of ions												
SuperClass	Classification information of the substance in the HMDB database												
Type	The matching mode includes MS2 forward/reverse matching (MS2 forward/reverse), first-order average molecular weight/single isotope molecular weight matching and no matching												
MEAN OO	The mean of the relative quantitative value of the substance in one of the experimental groups within the group comparison												
MEAN XX	The mean of the relative quantitative value of the substance in the other experimental group within the group comparison												
VIP	The substance in the group was compared to the OPLS-DA model obtained by the variable projection significance												
*P*-value	The *p*-value obtained by the *t*-test of this substance in the group comparison, *p*-value = probabilityof hypothesis being correct but rejected = number of negative results/total number of results, is a test probability against the sample data												
Q-value	The hypothesis test statistic (*p*-value) is the result after the correction of multiple hypothesis test. Q value = the probability of being rejected but correct = the number of false-positive results/thenumber of presumed positive results. It is a test probability of the inference obtained from statistical test and the re-statistics of *p*-value												
FOLD CHANGE	The substance in this group was compared with the multiple relationship between the two groups of experiments												
LOG_FOLDCHANGE	FOLD CHANGE takes the logarithm base 2												

**Table 2:** Differential metabolites detected by mass spectrometry combined with gas phase.

ID	Peak2	Similarity	rt	Count	Mass	MEAN T	MEAN M	VIP	*P*-value	*Q*-value	FOLDCHANGE	LOG_FOLDCHANGE
69	2-Hydroxybutanoic acid	923	5.90393,0	9	131	1.272515066	1.829157	1.299843399	0.030824766	0.054717746	0.695683796	−0.523496376
87	3-Hydroxybutyric acid	917	6.15207,0	9	147	25.48762597	38.1509	1.435772881	0.006906593	0.031058352	0.668074026	−0.581920125
228	L-malic acid	910	8.38126,0	9	73	15.02098304	24.18815	1.206850204	0.045844599	0.064707169	0.621005853	−0.68732123
407	Palmitoleic acid	903	11.4327,0	9	55	4.427715453	6.258029	1.236645983	0.049653209	0.067078325	0.707525543	−0.499145861
410	Palmitic acid	878	11.5306,0	9	117	20.90998716	28.73753	1.309850706	0.025056075	0.05026023	0.727619379	−0.458744127
68	Glycine 1	876	5.88319,0	9	102	3.920530889	5.969469	1.437149115	0.003623146	0.031058352	0.656763806	−0.606553473
144	glycerol	855	6.94956,0	9	205	14.73609113	22.11935	1.316507776	0.030374647	0.054407164	0.66620819	−0.585955005
234	Threitol	852	8.47978,0	9	217	0.320598289	0.534807	1.439750027	0.003284907	0.031058352	0.599465913	−0.738250374
273	Alpha-ketoglutaric acid	826	8.93667,0	9	73	4.392760756	7.040861	1.19046366	0.04958074	0.067035106	0.623895417	−0.680623882
172	Uracil	822	7.43667,0	9	241	0.382125402	0.661469	1.353053457	0.022060725	0.047455437	0.577691894	−0.791627843
184	Pelargonic acid	794	7.60111,0	9	215	0.046390864	0.07566	1.23255862	0.043826199	0.063362351	0.613151594	−0.705684289
222	Aminomalonic acid	791	8.28837,0	9	218	0.413476931	0.584889	1.244433147	0.031388031	0.055098631	0.706932665	−0.50035529
206	2-Deoxytetronic acid	780	8.01104,0	9	73	4.287775664	6.992805	1.380659529	0.007758549	0.031058352	0.613169609	−0.705641901
85	3-Hydroxypyridine	778	6.0817,0	9	152	0.233075193	0.349579	1.473437449	0.004190223	0.031058352	0.666731544	−0.584822111
127	4-Hydroxybutyrate	769	6.69919,0	9	233	0.018386599	0.032341	1.34064562	0.025110742	0.050307906	0.568527482	−0.814698006
427	heptadecanoic acid	745	11.985,0	9	117	0.110508121	0.168502	1.336703207	0.032082258	0.055556587	0.655828223	−0.608610108
197	Thymine	677	7.85356,0	9	270	0.043778381	0.066104	1.371605211	0.012906566	0.037801216	0.662263926	−0.59452182
110	Malonic acid 1	674	6.46348,0	9	141	0.120153186	0.212515	1.480974611	0.001576369	0.031058352	0.56538798	−0.822686883
99	Methyl phosphate	665	6.28585,0	9	241	0.115662474	0.211687	1.463936501	0.002727024	0.031058352	0.5463833	−0.872014707
207	Beta-alanine 2	643	8.03652,0	9	248	0.054056527	0.101364	1.421035491	0.014950627	0.039659996	0.533289189	−0.907010013
162	2,3-Dihydroxypyridine	633	7.28778,0	9	240	0.086432291	0.12972	1.393394351	0.006663844	0.031058352	0.666297891	−0.585760767
463	N-acetyl-5-hydroxytryptamine 1	590	13.2097,0	9	290	160.0062516	231.7193	1.303021085	0.041478731	0.061714361	0.690517501	−0.534250115
113	3-Aminoisobutyric acid 2	520	6.51726,0	9	57	0.32200107	0.496484	1.338967469	0.015637714	0.04020852	0.648562563	−0.624682345
307	1,2-Cyclohexanedione 4	517	9.492,0	8	205	0.101748813	0.181678	1.387571447	0.026616559	0.051576505	0.560051285	−0.836369152
216	Erythrose 1	514	8.18007,0	9	350	0.689931679	1.092036	1.272409468	0.028301472	0.052900868	0.631784782	−0.662494907
219	Capric acid	508	8.24689,0	9	117	0.161212023	0.274597	1.358543235	0.010727066	0.035339822	0.587086555	−0.768354878
146	2-Deoxyuridine	503	7.01356,0	3	81	0.000258002	0.659853	1.539040505	0.016123039	0.040576096	0.000390999	−11.32054852
377	D-altrose 2	487	10.8282,0	3	257	0.000258002	0.138383	1.541803731	0.000279156	0.031058352	0.0018644	−9.067072758
111	Methylmalonic acid	481	6.46941,0	9	226	0.210469269	0.423913	1.496235451	0.002203188	0.031058352	0.496491996	−1.010157634
285	Digitoxose 1	452	9.08156,0	3	73	0.000258002	0.000388	1.382297717	0.007898488	0.031058352	0.664566179	−0.589515221
246	trans-4-Hydroxy-L-proline 2	449	8.64244,0	3	186	0.000258002	0.000388	1.382297717	0.007898488	0.031058352	0.664566179	−0.589515221
88	Sulfuric acid	419	6.16896,0	9	281	0.759582216	1.140343	1.321189491	0.030078726	0.054199895	0.666099824	−0.586189695
218	2,4-Diaminobutyric acid 3	416	8.2337,0	9	103	0.177346978	0.279941	1.370608087	0.009107719	0.033074562	0.633516577	−0.658545725
425	d-Glucoheptose 1	412	11.9031,0	7	319	0.012258146	0.021077	1.315429516	0.039316399	0.060377704	0.581574962	−0.781962934
501	Isomaltose 2	410	15.118,0	7	73	0.21680148	0.330811	1.462708603	0.005347921	0.031058352	0.655363457	−0.609632863
211	N-acetyl-L-leucine 2	406	8.12433,0	8	86	0.058678762	0.087162	1.447579125	0.011125631	0.035834826	0.673218373	−0.570853545
309	L-homoserine 3	396	9.52289,0	6	290	0.052160115	0.075824	1.481796159	0.00209498	0.031058352	0.687909761	−0.539708769
77	2-Ketovaleric acid 2	393	5.97089,0	9	89	0.148017445	0.248358	1.44720369	0.004315057	0.031058352	0.595983832	−0.746654902
238	4-Acetamidobutyric acid 2	387	8.55919,0	9	174	0.262681494	0.414491	1.304395218	0.023923344	0.049245254	0.633744092	−0.658027703
343	Biuret 2	385	10.0571,0	6	171	0.131112438	0.11193	1.298491114	0.038783781	0.060035166	1.171379459	0.228208501
149	O-acetylserine 2	384	7.08467,0	3	116	0.000258002	0.000388	1.382297717	0.007898488	0.031058352	0.664566179	−0.589515221
170	Oxamide	382	7.37756,0	9	116	0.638063028	0.963743	1.375059784	0.010946865	0.035615566	0.66206751	−0.594949762
193	Resorcinol	364	7.74837,0	9	240	0.1893703	0.270561	1.422135343	0.028840031	0.053304636	0.699917794	−0.514742608
34	2-Ketobutyric acid 2	359	5.37696,0	9	174	3.895398713	6.310969	1.452753771	0.003819666	0.031058352	0.617242587	−0.69609049
495	Maltose	344	14.4673,0	3	204	0.000258002	0.000388	1.382297717	0.007898488	0.031058352	0.664566179	−0.589515221
506	Ergosterol	339	17.6037,0	7	129	0.038142477	0.170062	1.152056064	0.040476247	0.061105175	0.224285234	−2.156593449
290	D-erythronolactone 2	338	9.18748,0	9	244	0.233520567	0.335404	1.338741737	0.015238113	0.03989368	0.696237385	−0.522348811
417	Sinapyl alcohol 1	331	11.7691,0	3	241	0.000258002	0.000388	1.382297717	0.007898488	0.031058352	0.664566179	−0.589515221
416	N-acetyl-D-galactosamine 1	329	11.7478,0	3	202	0.000258002	0.000388	1.382297717	0.007898488	0.031058352	0.664566179	−0.589515221
114	3-Methylamino-1,2-propanediol 1	327	6.52733,0	9	116	0.145928713	0.251951	1.487710076	0.000575224	0.031058352	0.579194579	−0.787879996
92	4-Hydroxypyridine	326	6.20052,0	9	141	0.489927793	0.795927	1.451323971	0.002465951	0.031058352	0.615543839	−0.700066486
251	Cytosine	322	8.68941,0	9	314	2.509840552	4.082056	1.283159978	0.021963317	0.047357392	0.614847194	−0.701700187
507	Cholestane-3,5,6-triol, (3?5?6?-	321	18.7661,0	9	57	0.885146653	2.002186	1.387564892	0.006905578	0.031058352	0.442090187	−1.177587384
295	2-Carboxybenzaldehyde	318	9.29889,0	3	448	0.000258002	0.040566	1.54194144	0.001888566	0.031058352	0.006360115	−7.296731326
486	Alizarin	296	13.9349,0	3	192	0.000258002	0.000388	1.382297717	0.007898488	0.031058352	0.664566179	−0.589515221
255	Maleamate 2	272	8.72638,0	7	244	0.017984542	0.083238	1.133918922	0.023097427	0.048471018	0.216061427	−2.210486564
169	4-Acetylbutyric acid 2	265	7.37059,0	9	71	0.880439414	1.444854	1.436770641	0.018764895	0.043859407	0.60936233	−0.714627777
1	Analyte 1	0	4.85415,0	9	220	0.143627588	0.206994	1.412512528	0.009375416	0.03347991	0.693874841	−0.527252637
2	Analyte 2	0	4.86659,0	9	221	0.939179789	1.482562	1.223187422	0.048539846	0.066406466	0.633484237	−0.658619373
4	Analyte 4	0	4.906,0	9	127	25.04838891	40.41622	1.427277713	0.024022642	0.049336351	0.619760853	−0.690216464
9	Analyte 9	0	4.95133,0	9	56	0.076008724	0.130549	1.387105878	0.006481408	0.031058352	0.582225673	−0.780349639
13	Analyte 13	0	5.01281,0	9	281	0.519158543	0.88159	1.415956277	0.00432766	0.031058352	0.588888971	−0.763932441
14	Analyte 15	0	5.05415,0	9	89	0.126686366	0.194385	1.370732385	0.011314269	0.036061485	0.651727984	−0.617658153
15	Analyte 16	0	5.06096,0	9	71	0.14247583	0.254596	1.382415691	0.025128313	0.050323204	0.559614273	−0.837495336
16	Analyte 17	0	5.10467,0	9	248	0.189236239	0.277412	1.185862123	0.046349455	0.065033621	0.682149873	−0.551839351
17	Analyte 18	0	5.1217,0	9	71	0.23537597	0.369089	1.285237347	0.028223069	0.052841325	0.63772183	−0.649000828
21	Analyte 22	0	5.17593,0	9	160	0.117336438	0.227376	1.396764148	0.014713146	0.039462234	0.516045471	−0.954429902
26	Analyte 27	0	5.21607,0	9	207	1.534123014	2.442395	1.406790033	0.005116932	0.031058352	0.628122392	−0.670882394
28	Analyte 29	0	5.28378,0	9	318	0.490583082	0.81612	1.436795045	0.003319147	0.031058352	0.601116114	−0.7342844
29	Analyte 30	0	5.28985,0	9	141	0.424192495	0.699525	1.423841019	0.023362325	0.048722604	0.606400455	−0.72165726
31	Analyte 32	0	5.306,0	9	82	0.113890123	0.158043	1.300767064	0.041070167	0.061468223	0.720626315	−0.472676759
35	Analyte 36	0	5.38238,0	7	265	0.096659841	0.140997	1.323588558	0.026344832	0.051353772	0.685547745	−0.544670949
42	Analyte 43	0	5.50956,0	6	318	0.053147962	0.116636	1.389043774	0.004972626	0.031058352	0.45567417	−1.133925502
43	Analyte 44	0	5.53119,0	9	83	1.092646683	1.899617	1.374864683	0.014404096	0.039198218	0.575193161	−0.797881572
45	Analyte 46	0	5.57133,0	9	173	0.040681195	0.061956	1.280531443	0.032539528	0.055851534	0.656616292	−0.606877547
53	Analyte 54	0	5.69844,0	3	72	0.000258002	0.000388	1.382297717	0.007898488	0.031058352	0.664566179	−0.589515221
60	Analyte 61	0	5.77652,0	9	187	0.411739097	0.640732	1.381876488	0.034261264	0.056916913	0.642607665	−0.637989906
61	Analyte 62	0	5.78067,0	9	89	0.196207665	0.280779	1.249714074	0.032407158	0.055766689	0.698796716	−0.517055267
65	Analyte 66	0	5.85193,0	9	258	1.102739869	1.898571	1.45940324	0.002841114	0.031058352	0.58082638	−0.783821116
66	Analyte 67	0	5.86748,0	9	245	0.75141187	1.042003	1.340855538	0.014720908	0.039468767	0.721122764	−0.471683209
70	Analyte 71	0	5.91167,0	8	83	0.124600132	0.21066	1.460188	0.005923432	0.031058352	0.5914747	−0.757611636
71	Analyte 72	0	5.917,0	8	206	0.109884171	0.15268	1.26573403	0.041287114	0.061599284	0.719702949	−0.474526526
79	Analyte 80	0	5.97948,0	9	130	0.06545618	0.103826	1.457571818	0.002601319	0.031058352	0.630443919	−0.665560052
83	Analyte 84	0	6.06822,0	9	89	0.629883615	0.857153	1.348964908	0.02146227	0.046845666	0.734855621	−0.444467266
86	Analyte 87	0	6.1057,0	9	355	0.013100601	0.023343	1.464859492	0.002920262	0.031058352	0.561219926	−0.833361863
94	Analyte 95	0	6.23356,0	9	322	0.091050422	0.141299	1.335935817	0.013050372	0.037944549	0.644383212	−0.634009188
96	Analyte 97	0	6.25607,0	9	221	0.273334486	0.505144	1.382718281	0.018461158	0.043496426	0.541101922	−0.88602773
97	Analyte 98	0	6.26304,0	9	267	0.052183801	0.081353	1.339044648	0.021887851	0.047281114	0.641446738	−0.640598617
98	Analyte 99	0	6.28007,0	9	152	1.243281284	1.731247	1.362450345	0.018110888	0.04307052	0.718142143	−0.477658667
100	Analyte 101	0	6.35356,0	9	211	0.149975112	0.243234	1.269434425	0.034442556	0.057025121	0.6165884	−0.697620347
101	Analyte 102	0	6.37207,0	9	221	0.126068615	0.229719	1.369812092	0.014918915	0.039633839	0.548793895	−0.865663663
112	Analyte 114	0	6.51,0	3	207	0.000258002	0.000388	1.382297717	0.007898488	0.031058352	0.664566179	−0.589515221
124	Analyte 126	0	6.667,0	4	159	0.005980118	0.035398	1.206743852	0.007595819	0.031058352	0.168941731	−2.565402355
132	Analyte 134	0	6.76096,0	9	102	0.087156126	0.13135	1.424413383	0.004694475	0.031058352	0.663543315	−0.591737451
135	Analyte 137	0	6.83089,0	9	108	0.197475333	0.322281	1.394014594	0.009483719	0.033640096	0.612743258	−0.70664539
138	Analyte 140	0	6.86778,0	9	319	1.873157639	3.114122	1.418362299	0.007847303	0.031058352	0.601504164	−0.73335337
147	Analyte 149	0	7.02956,0	9	299	230.5117654	328.7347	1.368501132	0.014450612	0.039238447	0.70120909	−0.512083397
150	Analyte 152	0	7.08911,0	3	108	0.000258002	0.000388	1.382297717	0.007898488	0.031058352	0.664566179	−0.589515221
168	Analyte 170	0	7.36319,0	9	221	0.038675613	0.064306	1.425299204	0.003779737	0.031058352	0.601426746	−0.733539068
174	Analyte 176	0	7.44822,0	3	174	0.000258002	0.000388	1.382297717	0.007898488	0.031058352	0.664566179	−0.589515221
176	Analyte 178	0	7.4663,0	9	300	0.14066929	0.25431	1.397799484	0.009390264	0.033501999	0.553141309	−0.854280007
183	Analyte 185	0	7.57178,0	9	241	0.034659852	0.05353	1.278909492	0.022639121	0.048028239	0.647485316	−0.62708062
188	Analyte 190	0	7.65457,0	7	152	0.007184055	0.027339	1.117506783	0.048028223	0.066091974	0.262776377	−1.92809251
210	Analyte 212	0	8.07785,0	9	327	0.058009466	0.101589	1.381321063	0.008796901	0.032586355	0.571020535	−0.808385465
213	Analyte 215	0	8.15637,0	9	302	0.064257101	0.098722	1.246277713	0.028098654	0.052746433	0.650889175	−0.619516174
215	Analyte 217	0	8.16556,0	9	281	0.039345122	0.080491	1.374905261	0.003686217	0.031058352	0.48881664	−1.032634697
227	Analyte 229	0	8.36156,0	9	341	0.052448759	0.086946	1.325218816	0.017092886	0.041786174	0.603232561	−0.72921379
229	Analyte 231	0	8.40367,0	8	100	0.203180031	0.32939	1.341979958	0.013693148	0.038560536	0.616837794	−0.697036932
237	Analyte 240	0	8.54081,0	9	337	0.25036522	0.455655	1.366777246	0.036832395	0.058732065	0.549461995	−0.863908397
253	Analyte 256	0	8.70644,0	9	292	0.357528929	0.515931	1.21938121	0.048453083	0.066353391	0.692978537	−0.529117425
256	Analyte 259	0	8.74333,0	8	245	0.048845708	0.090829	1.326629434	0.009723922	0.033987829	0.537777275	−0.894919303
259	Analyte 262	0	8.78319,0	9	263	0.13088251	0.231069	1.344860566	0.044434442	0.063774445	0.566422421	−0.820049721
267	Analyte 270	0	8.88467,0	9	71	0.11227297	0.204138	1.433063266	0.00608667	0.031058352	0.549984911	−0.862536055
345	Analyte 350	0	10.0673,0	5	292	0.000258002	0.063722	1.539628212	0.004952684	0.031058352	0.004048846	−7.948273624
364	Analyte 369	0	10.4758,0	3	55	0.000258002	0.063552	1.542224923	0.016254112	0.040672706	0.004059669	−7.944422022
393	Analyte 400	0	11.1802,0	6	217	0.061713649	0.161587	1.405655109	0.013522845	0.038401138	0.38192255	−1.38864799
435	Analyte 442	0	12.219,0	4	67	0.005692675	0.030499	1.184497207	0.039424771	0.060446736	0.186653558	−2.421565088
455	Analyte 463	0	12.8955,0	9	232	3.442379815	4.609231	1.359547429	0.025385868	0.050546071	0.746844736	−0.421119747
487	Analyte 495	0	13.9389,0	6	259	0.013690852	0.027019	1.401570503	0.023138199	0.048509945	0.506703381	−0.98078664
503	Analyte 511	0	15.8464,0	9	357	0.080572597	0.145165	1.270859236	0.035617861	0.057880623	0.555041297	−0.849332977
ID	The unique data number of the substance in this qualitative analysis											
Peak	The name of the substance obtained by qualitative analysis											
Similarity	The matching degree between the substance and the substance in the standard library in qualitative analysis, and the value is [0, 1000]											
rt	Chromatographic retention time of the substance											
Count	The number of times the substance was detected in all experimental groups											
Mass	Material characteristics Mass charge ratio of ions											
MEAN OO	The mean of the relative quantitative value of the substance in one of the experimental groups within the group comparison											
MEAN XX	The mean of the relative quantitative value of the substance in the other experimental group within the group comparison											
VIP	The substance in the group was compared to the OPLS-DAmodel obtained by the variable projection significance											
*P*-value	The *p*-value obtained by the *t*-test of this substance in the group comparison, *p*-value = probability of hypothesis being correct but rejected = number of negative results/total number of results, is a test probability against the sample data											
*Q*-value	The hypothesis test statistic (*p*-value) is the result after the correction of multiple hypothesis test. Q-value = the probability of being rejected but correct = the number of false-positive results/the number of presumed positive results. It is a test probability of the inference obtained from statistical test and the re-statistics of *p*-value											
FOLD CHANGE	The substance in this group was compared with the multiple relationship between the two groups of experiments											
LOG_FOLDCHANGE	FOLD CHANGE takes the logarithm base 2											

**Table 3:** Enrichment analysis results based on mass spectrometry combined with HPLC.

Pathway	Total	Hits	Raw *p*	−ln (*p*)	Holm adjust	FDR	Impact	Hits cpd	Total cpd
Histidinemetabolism	15	2	0.011526	4.4632	0.95	0.94512	0.24194	L-Histidine cpd:C00135; carnosine cpd: C00386	L-glutamic acid cpd: C00025; 4-imidazolone-5-propionic acid cpd: C03680; urocanic acid cpd: C00785; L-histidine cpd: C00135; imidazole-4-acetaldehyde cpd: C05130; 1-methylhistamine cpd: C05127; methylimidazole acetaldehyde cpd: C05827; histamine cpd: C00388; carnosine cpd: C00386; N-formyl-L-aspartate cpd: C01044; formiminoglutamic acid cpd: C00439; imidazoleacetic acid cpd: C02835; methylimidazoleacetic acid cpd: C05828; L-aspartic acid cpd: C00049; 1-methylhistidine cpd: C01152
Aminoacyl-tRNA biosynthesis	69	3	0.039232	3.2383	1	1	0	L-histidine cpd: C00135; L-phenylalanine cpd: C00079; L-methionine cpd: C00073	L-asparagine cpd: C00152; tRNA(Asn) cpd: C01637; L-histidine cpd: C00135; tRNA(His) cpd: C01643; L-phenylalanine cpd: C00079; tRNA(Phe) cpd: C01648; L-arginine cpd: C00062; tRNA(Arg) cpd: C01636; L-glutamine cpd: C00064; tRNA(Gln) cpd: C01640; L-cysteine cpd: C00097; tRNA(Cys) cpd: C01639; glycine cpd: C00037; tRNA(Gly) cpd: C01642; tRNA(Asp) cpd: C01638; L-aspartic acid cpd: C00049; L-serine cpd: C00065; tRNA(Ser) cpd: C01650; L-methionine cpd: C00073; tRNA(Met) cpd: C01647; L-valine cpd: C00183; tRNA(Val) cpd: C01653; L-alanine cpd: C00041; tRNA(Ala) cpd: C01635; L-lysine cpd: C00047; tRNA(Lys) cpd: C01646; L-isoleucine cpd: C00407; tRNA(Ile) cpd: C01644; tRNA(Leu) cpd: C01645; L-leucine cpd: C00123; L-threonine cpd: C00188; tRNA(Thr) cpd: C01651; tRNA(Trp) cpd: C01652; L-tryptophan cpd: C00078; N10-formyl-thf cpd: C00234; L-methionyl-tRNA cpd: C02430; L-tyrosine cpd: C00082; tRNA(Tyr) cpd: C00787; L-proline cpd: C00148; tRNA(Pro) cpd: C01649; L-glutamic acid cpd: C00025; tRNA(Glu) cpd: C01641; glutaminyl-tRNA cpd: C02282; L-asparaginyl-tRNA(Asn) cpd: C03402; tRNA(Sec) cpd: C16636; L-seryl-tRNA(Sec) cpd: C06481; O-phosphoseryl-tRNA(Sec) cpd: C16638; L-histidyl-tRNA(His) cpd: C02988; L-phenylalanyl-tRNA(Phe) cpd: C03511; L-arginyl-tRNA(Arg) cpd: C02163; L-cysteinyl-tRNA(Cys) cpd: C03125; glycyl-tRNA(Gly) cpd: C02412; L-aspartyl-tRNA(Asp) cpd: C02984; L-seryl-tRNA(Ser) cpd: C02553; L-valyl-tRNA(Val) cpd: C02554; L-alanyl-tRNA cpd: C00886; L-lysyl-tRNA cpd: C01931; L-isoleucyl-tRNA(Ile) cpd: C03127; L-leucyl-tRNA cpd: C02047; L-threonyl-tRNA(Thr) cpd: C02992; L-tryptophanyl-tRNA(Trp) cpd: C03512; tetrahydrofolic acid cpd: C00101; N-formylmethionyl-tRNA cpd: C03294; L-tyrosyl-tRNA(Tyr) cpd: C02839; L-prolyl-tRNA(Pro) cpd: C02702; L-glutamyl-tRNA(Glu) cpd: C02987; L-glutamyl-tRNA(Gln) cpd: C06112; L-aspartyl-tRNA(Asn) cpd: C06113; L-selenocysteinyl-tRNA(Sec) cpd: C06482
Phenylalanine, tyrosine, and tryptophan biosynthesis	4	1	0.044453	3.1133	1	1	0.5	L-phenylalanine cpd: C00079	Phenylpyruvic acid cpd: C00166; L-phenylalanine cpd: C00079; L-tyrosine cpd: C00082; 4-hydroxyphenylpyruvic acid cpd: C01179
D-glutamine and D-glutamate metabolism	5	1	0.055273	2.8955	1	1	0	D-glutamine cpd: C00819	L-glutamic acid cpd: C00025; D-glutamine cpd: C00819; L-glutamine cpd: C00064; oxoglutaric acid cpd: C00026; D-glutamic acid cpd: C00217
Tryptophan metabolism	40	2	0.072666	2.6219	1	1	0.10987	L-kynurenine cpd: C00328; acetyl-N-formyl-5-methoxykynurenamine cpd: C05642	L-tryptophan cpd: C00078; melatonin cpd: C01598; serotonin cpd: C00780; 5-hydroxykynurenamine cpd: C05638; 5-hydroxykynurenine cpd: C05651; 5-hydroxy-L-tryptophan cpd: C00643; L-formylkynurenine cpd: C02700; acetoacetyl-CoA cpd: C00332; (S)-3-hydroxybutanoyl-CoA cpd: C01144; crotonoyl-CoA cpd: C00877; glutaryl-CoA cpd: C00527; oxoadipic acid cpd: C00322; 2-amino-3-carboxymuconic acid semialdehyde cpd: C04409; 3-hydroxyanthranilic acid cpd: C00632; L-kynurenine cpd: C00328; formylanthranilic acid cpd: C05653; L-3-hydroxykynurenine cpd: C03227; 3-hydroxykynurenamine cpd: C05636; indoleacetaldehyde cpd: C00637; 5-hydroxy-N-formylkynurenine cpd: C05648; 5-hydroxyindoleacetaldehyde cpd: C05634; tryptamine cpd: C00398; indoleacrylic acid cpd: C00331; acetyl-N-formyl-5-methoxykynurenamine cpd: C05642; 6-hydroxymelatonin cpd: C05643; N-acetylserotonin cpd: C00978; formyl-5-hydroxykynurenamine cpd: C05647; 4,6-dihydroxyquinoline cpd: C05639; acetyl-CoA cpd: C00024; 2-aminomuconic acid semialdehyde cpd: C03824; 2-aminobenzoic acid cpd: C00108; L-tryptophanyl-tRNA(Trp) cpd: C03512; cinnavalininate cpd: C05640; 4-(2-amino-3-hydroxyphenyl)-2,4-dioxobutanoic acid cpd: C05645; 4-(2-aminophenyl)-2,4-dioxobutanoic acid cpd: C01252; 4,8-dihydroxyquinoline cpd: C05637; indoleacetic acid cpd: C00954; 5-hydroxyindoleacetic acid cpd: C05635; N-methylserotonin cpd: C06212; N-methyltryptamine cpd: C06213
Nitrogen metabolism	9	1	0.097415	2.3288	1	1	0	L-histidine cpd: C00135	Ammonia cpd: C00014; carbon dioxide cpd: C00011; L-glutamic acid cpd: C00025; L-Glutamine cpd: C00064; L-cystathionine cpd: C02291; L-Histidine cpd: C00135; carbamoylphosphate cpd: C00169; carbonic acid cpd: C01353; glycine cpd: C00037
Phenylalanine metabolism	11	1	0.11782	2.1386	1	1	0.40741	L-phenylalanine cpd: C00079	Phenylacetaldehyde cpd: C00601; phenylacetyl-CoA cpd: C00582; L-phenylalanine cpd: C00079; phenylethylamine cpd: C05332; phenylpyruvic acid cpd: C00166; phenylacetic acid cpd: C07086; phenylacetylglycine cpd: C05598; ortho-hydroxyphenylacetic acid cpd: C05852; enol-phenylpyruvate cpd: C02763; 2-phenylacetamide cpd: C02505; L-tyrosine cpd: C00082
Sphingolipid metabolism	21	1	0.21351	1.5441	1	1	0	Phytosphingosine cpd: C12144	Sphinganine cpd: C00836; ceramide 1-phosphate cpd: C02960; sphingosine 1-phosphate cpd: C06124; sphinganine 1-phosphate cpd: C01120; dihydroceramide cpd: C12126; phytoceramide cpd: C12145; SM cpd: C00550; ceramide cpd: C00195; Palmityl-CoA cpd: C00154; L-Serine cpd: C00065; 3-dehydrosphinganine cpd: C02934; galabiosylceramide cpd: C06126; galactosylceramide cpd: C02686; GM4 cpd: C06128; lactosylceramide cpd: C01290; glucosylceramide cpd: C01190; sphingosine cpd: C00319; 3-O-Sulfogalactosylceramide (d18 : : 1/24 : : 0) cpd: C06125; phytosphingosine cpd: C12144; digalactosylceramidesulfate cpd: C06127; O-phosphoethanolamine cpd: C00346
Cysteine and methionine metabolism	27	1	0.26616	1.3236	1	1	0.08685	L-methionine cpd: C00073	2-Oxo-4-methylthiobutanoic acid cpd: C01180; 1,2-dihydroxy-3-keto-5-methylthiopentene cpd: C15606; 5′-methylthioadenosine cpd: C00170; S-adenosylmethioninamine cpd: C01137; S-adenosylmethionine cpd: C00019; L-cystathionine cpd: C02291; L-homocysteine cpd: C00155; L-serine cpd: C00065; L-methionine cpd: C00073; S-adenosylhomocysteine cpd: C00021; 2,3-diketo-5-methylthiopentyl-1-phosphate cpd: C15650; cysteic acid cpd: C00506; L-cystine cpd: C00491; L-cysteine cpd: C00097; 3-sulfinoalanine cpd: C00606; sulfite cpd: C00094; 3-mercaptopyruvic acid cpd: C00957; 3-methylthiopropionic acid cpd: C08276; 5-methylthioribose 1-phosphate cpd: C04188; 2-ketobutyric acid cpd: C00109; 2-aminoacrylic acid cpd: C02218; 3-sulfopyruvic acid cpd: C05528; thiocysteine cpd: C01962; 3-sulfinylpyruvic acid cpd: C05527; pyruvic acid cpd: C00022; thiosulfate cpd: C00320; 3-mercaptolactic acid cpd: C05823
Glycerophospholipid metabolism	30	1	0.29123	1.2336	1	1	0.02315	Glycerophosphocholine cpd: C00670	Phosphatidylethanolamine cpd: C00350; phosphatidylcholine cpd: C00157; dihydroxyacetone phosphate cpd: C00111; LysoPC (18 : 1(9Z)) cpd: C04230; 1,2-diacyl-sn-glycerol cpd: C00641; citicoline cpd: C00307; phosphorylcholine cpd: C00588; choline cpd: C00114; acetylcholine cpd: C01996; O-phosphoethanolamine cpd: C00346; ethanolamine cpd: C00189; PA (16 : : 0/16 : : 0) cpd: C00416; acyl-CoA cpd: C00040; 1-acyl-sn-glycerol 3-phosphate cpd: C00681; CDP-diacylglycerol cpd: C00269; glycerol 3-phosphate cpd: C00093; 1-acyl-sn-glycero-3-phosphoethanolamine cpd: C04438; 2-acyl-sn-glycero-3-phosphoethanolamine cpd: C05973; 2-acyl-sn-glycero-3-phosphocholine cpd: C04233; CDP-glycerol cpd: C00513; PS (16 : 0/16 : 0) cpd: C02737; phosphatidylglycerol cpd: C00344; dihydroxyacetone phosphate acyl ester cpd: C03372; CDP-ethanolamine cpd: C00570; 1-phosphatidyl-D-myo-inositol cpd: C01194; glycerylphosphorylethanolamine cpd: C01233; glycerophosphocholine cpd: C00670; phosphatidyl-N-methylethanolamine cpd: C01241; phosphatidylglycerophosphate cpd: C03892; cardiolipin cpd: C05980
Arginine and proline metabolism	44	1	0.39796	0.9214	1	1	0	N-acetylornithine cpd: C00437	L-glutamine cpd: C00064; ammonia cpd: C00014; carbamoylphosphate cpd: C00169; ornithine cpd: C00077; citrulline cpd: C00327; L-aspartic acid cpd: C00049; argininosuccinic acid cpd: C03406; L-arginine cpd: C00062; L-glutamic acid cpd: C00025; N-acetylornithine cpd: C00437; L-proline cpd: C00148; peptide cpd: C00012; D-proline cpd: C00763; hydroxyproline cpd: C01157; pyrroline hydroxycarboxylic acid cpd: C04281; L-4-hydroxyglutamate semialdehyde cpd: C05938; L-erythro-4-hydroxyglutamate cpd: C05947; N-(o)-hydroxyarginine cpd: C05933; guanidinoacetic acid cpd: C00581; creatine cpd: C00300; gamma-aminobutyric acid cpd: C00334; agmatine cpd: C00179; L-glutamic-gamma-semialdehyde cpd: C01165; L-glutamic acid 5-phosphate cpd: C03287; (S)-1-pyrroline-5-carboxylate cpd: C03912; putrescine cpd: C00134; 4-aminobutyraldehyde cpd: C00555; S-adenosylmethioninamine cpd: C01137; S-adenosylmethionine cpd: C00019; spermidine cpd: C00315; N-acetylputrescine cpd: C02714; N4-acetylaminobutanal cpd: C05936; cis-4-hydroxy-D-proline cpd: C03440; fumaric acid cpd: C00122; urea cpd: C00086; N-acetyl-L-alanine cpd: C00624; 1-pyrroline-2-carboxylic acid cpd: C03564; D-4-hydroxy-2-oxoglutarate cpd: C05946; nitric oxide cpd: C00533; phosphocreatine cpd: C02305; 4-guanidinobutanoic acid cpd: C01035; spermine cpd: C00750; 4-acetamidobutanoic acid cpd: C02946; 1-pyrroline-4-hydroxy-2-carboxylate cpd: C04282
Pathway	Metabolic pathway name								
Total	The number of metabolites in this pathway								
Hits	The number of differential metabolites hit this pathway								
Raw *p*	*P* value of metabolic pathway enrichment analysis								
−ln(p)	Minus log base E of P								
Holm adjust	*P* values corrected by Holm–Bonferroni method for multiple hypothesis testing								
FDR	*P* value corrected by false discovery rate (FDR) method for multiple hypothesis testing								
Impact	Impact value of metabolic pathway topology analysis								
Hits cpd	The names and KEGG IDs of differential metabolites hit the pathway								
Total cpd	All metabolite names and KEGG IDs contained in this pathway								

**Table 4:** Enrichment analysis results based on mass spectrometry combined with gas phase.

Pathway	Total	Hits	Raw *p*	−ln(*p*)	Holm adjust	FDR	Impact	Hits cpd	Total cpd
Pyrimidine metabolism	41	5	0.0013	6.6293	0.11	0.108	0.09731	Deoxyuridine cpd: C00526; uracil cpd: C00106; beta-alanine cpd: C00099; thymine cpd: C00178; 3-aminoisobutanoic acid cpd: C05145	Thioredoxin cpd: C00342; uridine 5′-diphosphate cpd: C00015; L-Glutamine cpd: C00064; carbamoylphosphate cpd: C00169; 4,5-dihydroorotic acid cpd: C00337; orotidylic acid cpd: C01103; RNA cpd: C00046; uridine triphosphate cpd: C00075; uridine 5′-monophosphate cpd: C00105; uridine cpd: C00299; dihydrouracil cpd: C00429; ureidopropionic acid cpd: C02642; cytidine triphosphate cpd: C00063; CDP cpd: C00112; cytidine monophosphate cpd: C00055; cytidine cpd: C00475; thioredoxin disulfide cpd: C00343; dCDP cpd: C00705; dCTP cpd: C00458; dCMP cpd: C00239; deoxycytidine cpd: C00881; deoxyuridine triphosphate cpd: C00460; dUDP cpd: C01346; dUMP cpd: C00365; deoxyuridine cpd: C00526; thymidine 5′-triphosphate cpd: C00459; dTDP cpd: C00363; 5-thymidylic acid cpd: C00364; thymidine cpd: C00214; dihydrothymine cpd: C00906; ureidoisobutyric acid cpd: C05100; P1,P4-bis(5′-uridyl) tetraphosphate cpd: C06198; ureidosuccinic acid cpd: C00438; phosphoribosyl pyrophosphate cpd: C00119; orotic acid cpd: C00295; uracil cpd: C00106; beta-alanine cpd: C00099; DNA cpd: C00039; deoxyribose 1-phosphate cpd: C00672; thymine cpd: C00178; 3-aminoisobutanoic acid cpd: C05145
Pantothenate and CoA biosynthesis	15	2	0.0384	3.2608	1	1	0	Beta-alanine cpd: C00099; uracil cpd: C00106	Dephospho-CoA cpd: C00882; pantetheine 4′-phosphate cpd: C01134; pantetheine cpd: C00831; 4-phosphopantothenoylcysteine cpd: C04352; D-pantothenoyl-L-cysteine cpd: C04079; d-4′-phosphopantothenate cpd: C03492; L-cysteine cpd: C00097; pantothenic acid cpd: C00864; ureidopropionic acid cpd: C02642; dihydrouracil cpd: C00429; L-valine cpd: C00183; coenzyme a cpd: C00010; beta-alanine cpd: C00099; uracil cpd: C00106; alpha-ketoisovaleric acid cpd: C00141
Beta-alanine metabolism	17	2	0.0484	3.0283	1	1	0.44444	Beta-alanine cpd: C00099; uracil cpd: C00106	Acrylyl-CoA cpd: C00894; 3-hydroxypropionyl-CoA cpd: C05668; hydroxypropionic acid cpd: C01013; malonyl-CoA cpd: C00083; beta-alanine cpd: C00099; L-aspartic acid cpd: C00049; spermine cpd: C00750; 1,3-diaminopropane cpd: C00986; 3-aminopropionaldehyde cpd: C05665; ureidopropionic acid cpd: C02642; dihydrouracil cpd: C00429; anserine cpd: C01262; propionyl-CoA cpd: C00100; Acetyl-CoA cpd: C00024; malonic semialdehyde cpd: C00222; spermidine cpd: C00315; uracil cpd: C00106
Starch and sucrose metabolism	19	2	0.0593	2.8253	1	1	0.07451	D-maltose cpd: C00208; isomaltose cpd: C00252	beta-D-fructose cpd: C02336; sucrose cpd: C00089; uridine diphosphate glucuronic acid cpd: C00167; uridine diphosphate glucose cpd: C00029; glucose 1-phosphate cpd: C00103; 3-methoxy-4-hydroxyphenylglycol glucuronide cpd: C03033; glucose 6-phosphate cpd: C00668; alpha-D-glucose cpd: C00267; starch cpd: C00369; amylose cpd: C00718; trehalose cpd: C01083; D-maltose cpd: C00208; D-glucose cpd: C00031; dextrin cpd: C00721; isomaltose cpd: C00252; beta-D-fructose 6-phosphate cpd: C05345; UDP-D-xylose cpd: C00190; alpha-D-glucose 1,6-bisphosphate cpd: C01231; D-glucuronic acid cpd: C00191
Propanoate metabolism	20	2	0.065	2.733	1	1	0	Beta-alanine cpd: C00099; 2-hydroxybutyric acid cpd: C05984	(S)-methylmalonic acid semialdehyde cpd: C06002; methylmalonyl-CoA cpd: C00683; propionyl-CoA cpd: C00100; propinol adenylate cpd: C05983; R-methylmalonyl-CoA cpd: C01213; succinic acid cpd: C00042; hydroxypropionic acid cpd: C01013; beta-alanine cpd: C00099; 2-propyn-1-al cpd: C05985; acetyl-CoA cpd: C00024; malonyl-CoA cpd: C00083; 2-hydroxybutyric acid cpd: C05984; acrylyl-CoA cpd: C00894; propionic acid cpd: C00163; succinyl-CoA cpd: C00091; 3-hydroxypropionyl-CoA cpd: C05668; malonic semialdehyde cpd: C00222; propynoic acid cpd: C00804; acetoacetyl-CoA cpd: C00332; 2-ketobutyric acid cpd: C00109
D-glutamine and D-glutamate metabolism	5	1	0.1016	2.2866	1	1	0	Oxoglutaric acid cpd: C00026	L-glutamic acid cpd: C00025; D-glutamine cpd: C00819; L-glutamine cpd: C00064; oxoglutaric acid cpd: C00026; D-glutamic acid cpd: C00217
Sulfur metabolism	5	1	0.1016	2.2866	1	1	0.3	Sulfate cpd: C00059	Phosphoadenosine phosphosulfate cpd: C00053; adenosine phosphosulfate cpd: C00224; sulfite cpd: C00094; sulfate cpd: C00059; adenosine 3′,5′-diphosphate cpd: C00054
Cyanoamino acid metabolism	6	1	0.1207	2.1145	1	1	0	Glycine cpd: C00037	3-Cyano-L-alanine cpd: C02512; beta-aminopropionitrile cpd: C05670; glycine cpd: C00037; gamma-glutamyl-beta-cyanoalanine cpd: C05711; gamma-glutamyl-beta-aminopropiononitrile cpd: C06114; L-serine cpd: C00065
Methane metabolism	9	1	0.1756	1.7394	1	1	0	Glycine cpd: C00037	S-formylglutathione cpd: C01031; glycine cpd: C00037; 5,10-methylene-THF cpd: C00143; S-(Hydroxymethyl)glutathione cpd: C14180; methanol cpd: C00132; formic acid cpd: C00058; L-serine cpd: C00065; formaldehyde cpd: C00067; 5-methyltetrahydrofolic acid cpd: C00440
Nitrogen metabolism	9	1	0.1756	1.7394	1	1	0	Glycine cpd: C00037	Ammonia cpd: C00014; carbon dioxide cpd: C00011; L-glutamic acid cpd: C00025; L-glutamine cpd: C00064; L-cystathionine cpd: C02291; L-histidine cpd: C00135; carbamoylphosphate cpd: C00169; carbonic acid cpd: C01353; glycine cpd: C00037
Arginine and proline metabolism	44	2	0.2382	1.4345	1	1	0.04414	Hydroxyproline cpd: C01157; 4-acetamidobutanoic acid cpd: C02946	L-glutamine cpd: C00064; ammonia cpd: C00014; carbamoylphosphate cpd: C00169; ornithine cpd: C00077; citrulline cpd: C00327; L-aspartic acid cpd: C00049; argininosuccinic acid cpd: C03406; L-arginine cpd: C00062; L-glutamic acid cpd: C00025; N-acetylornithine cpd: C00437; L-proline cpd: C00148; peptide cpd: C00012; D-proline cpd: C00763; hydroxyproline cpd: C01157; pyrroline hydroxycarboxylic acid cpd: C04281; L-4-hydroxyglutamate semialdehyde cpd: C05938; L-erythro-4-hydroxyglutamate cpd: C05947; N-(o)-hydroxyarginine cpd: C05933; guanidinoacetic acid cpd: C00581; creatine cpd: C00300; gamma-aminobutyric acid cpd: C00334; agmatine cpd: C00179; L-glutamic-gamma-semialdehyde cpd: C01165; L-glutamic acid 5-phosphate cpd: C03287; (S)-1-pyrroline-5-carboxylate cpd: C03912; putrescine cpd: C00134; 4-aminobutyraldehyde cpd: C00555; S-adenosylmethioninamine cpd: C01137; S-adenosylmethionine cpd: C00019; spermidine cpd: C00315; N-Acetylputrescine cpd: C02714; N4-Acetylaminobutanal cpd: C05936; cis-4-hydroxy-D-proline cpd: C03440; Fumaric acid cpd: C00122; urea cpd: C00086; N-Acetyl-L-alanine cpd: C00624; 1-pyrroline-2-carboxylic acid cpd: C03564; D-4-hydroxy-2-oxoglutarate cpd: C05946; nitric oxide cpd: C00533; phosphocreatine cpd: C02305; 4-guanidinobutanoic acid cpd: C01035; spermine cpd: C00750; 4-acetamidobutanoic acid cpd: C02946; 1-pyrroline-4-hydroxy-2-carboxylate cpd: C04282
Glycerolipid metabolism	18	1	0.3213	1.1355	1	1	0.28098	Glycerol cpd: C00116	Triacylglycerol cpd: C00422; acyl-CoA cpd: C00040; 1,2-diacyl-sn-glycerol cpd: C00641; PA(16 : : 0/16 : : 0) cpd: C00416; 1-acyl-sn-glycerol 3-phosphate cpd: C00681; 1-acylglycerol cpd: C01885; glycerol 3-phosphate cpd: C00093; dihydroxyacetone cpd: C00184; glycerol cpd: C00116; lactaldehyde cpd: C05999; D-glyceraldehyde cpd: C00577; glyceric acid cpd: C00258; digalactosyl-diacylglycerol cpd: C06037; fatty acid cpd: C00162; dihydroxyacetone phosphate cpd: C00111; propylene glycol cpd: C00583; 3-phospho-D-glycerate cpd: C00197; 1,2-diacyl-3-beta-D-galactosyl-sn-glycerol cpd: C03692
Citrate cycle (TCA cycle)	20	1	0.3501	1.0496	1	1	0.06799	Oxoglutaric acid cpd: C00026	Enzyme N6-(dihydrolipoyl)lysine cpd: C15973; oxoglutaric acid cpd: C00026; thiamine pyrophosphate cpd: C00068; Enzyme N6-(lipoyl)lysine cpd: C15972; 3-carboxy-1-hydroxypropylthiamine diphosphate cpd: C05381; Succinyl-CoA cpd: C00091; succinic acid cpd: C00042; oxalosuccinic acid cpd: C05379; isocitric acid cpd: C00311; oxalacetic acid cpd: C00036; Acetyl-CoA cpd: C00024; L-malic acid cpd: C00149; cis-aconitic acid cpd: C00417; citric acid cpd: C00158; pyruvic acid cpd: C00022; 2-(a-hydroxyethyl)thiamine diphosphate cpd: C05125; [dihydrolipoyllysine-residue succinyltransferase] S-succinyldihydrolipoyllysine cpd: C16254; Fumaric acid cpd: C00122; S-acetyldihydrolipoamide-E cpd: C16255; phosphoenolpyruvic acid cpd: C00074
Butanoate metabolism	22	1	0.3777	0.97365	1	1	0	Oxoglutaric acid cpd: C00026	3-Butyn-1-al cpd: C06145; (R)-3-hydroxybutyric acid cpd: C01089; acetoacetic acid cpd: C00164; 3-hydroxy-3-methylglutaryl-CoA cpd: C00356; Acetyl-CoA cpd: C00024; Acetoacetyl-CoA cpd: C00332; (S)-3-hydroxybutanoyl-CoA cpd: C01144; Crotonoyl-CoA cpd: C00877; gamma-aminobutyric acid cpd: C00334; L-glutamic acid cpd: C00025; Butanoyl-CoA cpd: C00136; butanal cpd: C01412; succinic acid semialdehyde cpd: C00232; butyric acid cpd: C00246; oxoglutaric acid cpd: C00026; thiamine pyrophosphate cpd: C00068; pyruvic acid cpd: C00022; 3-butynoate cpd: C06144; 1-butanol cpd: C06142; succinic acid cpd: C00042; 2-hydroxyglutarate cpd: C02630; 2-(a-hydroxyethyl)thiamine diphosphate cpd: C05125
Alanine, aspartate, and glutamate metabolism	24	1	0.4042	0.90587	1	1	0.06329	Oxoglutaric acid cpd: C00026	N-Acetyl-L-aspartic acid cpd: C01042; 2-oxosuccinamate cpd: C02362; L-aspartic acid cpd: C00049; D-aspartic acid cpd: C00402; argininosuccinic acid cpd: C03406; adenylsuccinic acid cpd: C03794; L-Alanine cpd: C00041; succinic acid semialdehyde cpd: C00232; L-glutamic acid cpd: C00025; gamma-aminobutyric acid cpd: C00334; L-Glutamine cpd: C00064; ammonia cpd: C00014; 2-keto-glutaramic acid cpd: C00940; (S)-1-pyrroline-5-carboxylate cpd: C03912; oxalacetic acid cpd: C00036; L-Asparagine cpd: C00152; Fumaric acid cpd: C00122; pyruvic acid cpd: C00022; ureidosuccinic acid cpd: C00438; succinic acid cpd: C00042; oxoglutaric acid cpd: C00026; carbamoylphosphate cpd: C00169; glucosamine 6-phosphate cpd: C00352; 5-phosphoribosylamine cpd: C03090
Galactose metabolism	26	1	0.4296	0.84494	1	1	0	Glycerol cpd: C00116	Stachyose cpd: C01613; D-tagatose 6-phosphate cpd: C01097; D-gal alpha 1->6d-gal alpha 1->6d-glucose cpd: C05404; sucrose cpd: C00089; raffinose cpd: C00492; melibiose cpd: C05402; D-galactose cpd: C00124; galactosylglycerol cpd: C05401; epimelibiose cpd: C05400; melibiitol cpd: C05399; galactinol cpd: C01235; Alpha-D-Glucose cpd: C00267; alpha-lactose cpd: C00243; glucose 1-phosphate cpd: C00103; uridine diphosphategalactose cpd: C00052; uridine diphosphate glucose cpd: C00029; galactose 1-phosphate cpd: C00446; glucose 6-phosphate cpd: C00668; D-tagatose 1,6-bisphosphate cpd: C03785; D-Fructose cpd: C00095; D-glucose cpd: C00031; galactitol cpd: C01697; glycerol cpd: C00116; D-Mannose cpd: C00159; sorbitol cpd: C00794; myoinositol cpd: C00137
Glutathione metabolism	26	1	0.4296	0.84494	1	1	0.00573	Glycine cpd: C00037	R-S-cysteinylglycine cpd: C05729; R-S-glutathione cpd: C02320; glutathione cpd: C00051; NADP cpd: C00006; NADPH cpd: C00005; oxidized glutathione cpd: C00127; gamma-glutamylcysteine cpd: C00669; glycine cpd: C00037; L-cysteine cpd: C00097; L-glutamic acid cpd: C00025; cysteinylglycine cpd: C01419; pyroglutamic acid cpd: C01879; L-amino acid cpd: C00151; 5-L-glutamyl-L-alanine cpd: C03740; RX cpd: C01322; ornithine cpd: C00077; putrescine cpd: C00134; spermidine cpd: C00315; cadaverine cpd: C01672; tryparedoxin cpd: C16663; trypanothione cpd: C02090; S-substituted L-cysteine cpd: C05726; spermine cpd: C00750; aminopropylcadaverine cpd: C16565; tryparedoxin disulfide cpd: C16664; trypanothione disulfide cpd: C03170
Fatty acid elongation in mitochondria	27	1	0.4419	0.8167	1	1	0	Palmitic acid cpd: C00249	Palmityl-CoA cpd: C00154; (2e)-hexadecenoyl-CoA cpd: C05272; (S)-3-hydroxyhexadecanoyl-CoA cpd: C05258; 3-oxohexadecanoyl-CoA cpd: C05259; Acetyl-CoA cpd: C00024; Tetradecanoyl-CoA cpd: C02593; (2e)-tetradecenoyl-CoA cpd: C05273; (S)-3-hydroxytetradecanoyl-CoA cpd: C05260; 3-oxotetradecanoyl-CoA cpd: C05261; Lauroyl-CoA cpd: C01832; (2e)-dodecenoyl-CoA cpd: C03221; (S)-3-hydroxydodecanoyl-CoA cpd: C05262; 3-oxododecanoyl-CoA cpd: C05263; Decanoyl-CoA (n-C10: 0CoA) cpd: C05274; (2e)-decenoyl-CoA cpd: C05275; (S)-hydroxydecanoyl-CoA cpd: C05264; 3-oxodecanoyl-CoA cpd: C05265; Octanoyl-CoA cpd: C01944; (2e)-octenoyl-CoA cpd: C05276; (S)-hydroxyoctanoyl-CoA cpd: C05266; 3-oxooctanoyl-CoA cpd: C05267; Hexanoyl-CoA cpd: C05270; trans-2-hexenoyl-CoA cpd: C05271; (S)-hydroxyhexanoyl-CoA cpd: C05268; 3-oxohexanoyl-CoA cpd: C05269; Butanoyl-CoA cpd: C00136; palmitic acid cpd: C00249
Porphyrin and chlorophyll metabolism	27	1	0.4419	0.8167	1	1	0	Glycine cpd: C00037	Cobinamide cpd: C05774; heme O cpd: C15672; heme cpd: C00032; glycine cpd: C00037; bilirubin diglucuronide cpd: C05787; Cob(I)yrinate a,c diamide cpd: C06505; Fe2 + cpd: C14818; hemoglobin cpd: C01708; biliverdin cpd: C00500; protoporphyrinogen IX cpd: C01079; coproporphyrin III cpd: C03263; uroporphyrinogen III cpd: C01051; hydroxymethylbilane cpd: C01024; porphobilinogen cpd: C00931; 5-aminolevulinic acid cpd: C00430; uroporphyrinogen I cpd: C05766; L-Glutamic acid cpd: C00025; bilirubin cpd: C00486; protoporphyrin IX cpd: C02191; adenosyl cobinamide cpd: C06508; heme a cpd: C15670; D-Urobilinogen cpd: C05791; adenosyl cobyrinic acid a,c diamide cpd: C06506; Fe3+ cpd: C14819; coproporphyrinogen I cpd: C05768; l-glutamyl-tRNA(Glu) cpd: C02987; cytochrome c cpd: C00524
Glycine, serine, and threonine metabolism	31	1	0.4886	0.71625	1	1	0.26884	Glycine cpd: C00037	L-Serine cpd: C00065; choline cpd: C00114; glyceric acid cpd: C00258; betaine cpd: C00719; guanidinoacetic acid cpd: C00581; 3-phospho-D-glycerate cpd: C00197; dimethylglycine cpd: C01026; L-cystathionine cpd: C02291; glycine cpd: C00037; phosphoserine cpd: C01005; sarcosine cpd: C00213; 5,10-methylene-THF cpd: C00143; L-threonine cpd: C00188; lipoylprotein cpd: C02051; aminoacetone cpd: C01888; tetrahydrofolic acid cpd: C00101; S-aminomethyldihydrolipoylprotein cpd: C01242; dihydrolipoylprotein cpd: C02972; D-serine cpd: C00740; betaine aldehyde cpd: C00576; hydroxypyruvic acid cpd: C00168; creatine cpd: C00300; phosphohydroxypyruvic acid cpd: C03232; L-cysteine cpd: C00097; glyoxylic acid cpd: C00048; L-2-amino-3-oxobutanoic acid cpd: C03508; pyruvic acid cpd: C00022; carbon dioxide cpd: C00011; 5-aminolevulinic acid cpd: C00430; pyruvaldehyde cpd: C00546; ammonia cpd: C00014
Valine, leucine, and isoleucine degradation	38	1	0.5614	0.57739	1	1	0	Methylmalonic acid cpd: C02170	Enzyme N6-(lipoyl)lysine cpd: C15972; 2-methyl-1-hydroxybutyl-ThPP cpd: C15978; enzyme N6-(dihydrolipoyl)lysine cpd: C15973; 2-methyl-1-hydroxypropyl-ThPP cpd: C15976; 3-methyl-1-hydroxybutyl-ThPP cpd: C15974; acetyl-CoA cpd: C00024; acetoacetyl-CoA cpd: C00332; acetoacetic acid cpd: C00164; 3-hydroxy-3-methylglutaryl-CoA cpd: C00356; 3-methylcrotonyl-CoA cpd: C03069; 3-hydroxyisovaleryl-CoA cpd: C05998; isovaleryl-CoA cpd: C02939; 3-methyl-2-oxovaleric acid cpd: C00671; thiamine pyrophosphate cpd: C00068; L-valine cpd: C00183; 2-methylacetoacetyl-CoA cpd: C03344; (S)-3-hydroxyisobutyrate cpd: C06001; tiglyl-CoA cpd: C03345; butyryl-CoA cpd: C00630; S-(2-methylbutanoyl)-dihydrolipoamide cpd: C15979; alpha-ketoisovaleric acid cpd: C00141; L-isoleucine cpd: C00407; R-methylmalonyl-CoA cpd: C01213; methylmalonyl-CoA cpd: C00683; propionyl-CoA cpd: C00100; (S)-methylmalonic acid semialdehyde cpd: C06002; (S)-b-aminoisobutyric acid cpd: C03284; 2-methyl-3-hydroxybutyryl-CoA cpd: C04405; (S)-3-hydroxyisobutyryl-CoA cpd: C06000; methacrylyl-CoA cpd: C03460; (S)-2-methylbutanoyl-CoA cpd: C15980; S-(2-methylpropionyl)-dihydrolipoamide-E cpd: C15977; 4-methyl-2-oxopentanoate cpd: C00233; S-(3-methylbutanoyl)-dihydrolipoamide-E cpd: C15975; L-leucine cpd: C00123; 3-methylglutaconyl-CoA cpd: C03231; succinyl-CoA cpd: C00091; methylmalonic acid cpd: C02170
Fatty acid metabolism	39	1	0.5709	0.56053	1	1	0	Palmitic acid cpd: C00249	Palmityl-CoA cpd: C00154; hexanoyl-CoA cpd: C05270; butanoyl-CoA cpd: C00136; acetyl-CoA cpd: C00024; (S)-3-hydroxybutanoyl-CoA cpd: C01144; cis,cis-3,6-dodecadienoyl-CoA cpd: C05280; (S)-hydroxyhexanoyl-CoA cpd: C05268; (S)-hydroxyoctanoyl-CoA cpd: C05266; octanoyl-CoA cpd: C01944; (S)-hydroxydecanoyl-CoA cpd: C05264; decanoyl-CoA (n-C10: 0CoA) cpd: C05274; (S)-3-hydroxydodecanoyl-CoA cpd: C05262; lauroyl-CoA cpd: C01832; primary alcohol cpd: C00226; glutaryl-CoA cpd: C00527; (S)-3-hydroxytetradecanoyl-CoA cpd: C05260; tetradecanoyl-CoA cpd: C02593; fatty acid cpd: C00162; (S)-3-hydroxyhexadecanoyl-CoA cpd: C05258; palmitic acid cpd: C00249; (2E)-hexadecenoyl-CoA cpd: C05272; trans-2-hexenoyl-CoA cpd: C05271; crotonoyl-CoA cpd: C00877; acetoacetyl-CoA cpd: C00332; trans,cis-Lauro-2,6-dienoyl-CoA cpd: C05279; coenzyme A cpd: C00010; 3-oxohexanoyl-CoA cpd: C05269; 3-oxooctanoyl-CoA cpd: C05267; (2E)-octenoyl-CoA cpd: C05276; 3-oxodecanoyl-CoA cpd: C05265; (2E)-decenoyl-CoA cpd: C05275; 3-oxododecanoyl-CoA cpd: C05263; (2E)-dodecenoyl-CoA cpd: C03221; aldehyde cpd: C00071; 3-oxotetradecanoyl-CoA cpd: C05261; (2E)-tetradecenoyl-CoA cpd: C05273; omega-hydroxy fatty acid cpd: C03547; 3-oxohexadecanoyl-CoA cpd: C05259; L-palmitoylcarnitine cpd: C02990
Tryptophan metabolism	40	1	0.5803	0.5443	1	1	0	N-acetylserotonin cpd: C00978	L-tryptophan cpd: C00078; melatonin cpd: C01598; serotonin cpd: C00780; 5-hydroxykynurenamine cpd: C05638; 5-hydroxykynurenine cpd: C05651; 5-hydroxy-L-tryptophan cpd: C00643; L-formylkynurenine cpd: C02700; acetoacetyl-CoA cpd: C00332; (S)-3-hydroxybutanoyl-CoA cpd: C01144; crotonoyl-CoA cpd: C00877; glutaryl-CoA cpd: C00527; oxoadipic acid cpd: C00322; 2-amino-3-carboxymuconic acid semialdehyde cpd: C04409; 3-hydroxyanthranilic acid cpd: C00632; L-kynurenine cpd: C00328; formylanthranilic acid cpd: C05653; L-3-hydroxykynurenine cpd: C03227; 3-hydroxykynurenamine cpd: C05636; indoleacetaldehyde cpd: C00637; 5-hydroxy-N-formylkynurenine cpd: C05648; 5-hydroxyindoleacetaldehyde cpd: C05634; tryptamine cpd: C00398; indoleacrylic acid cpd: C00331; acetyl-N-formyl-5-methoxykynurenamine cpd: C05642; 6-hydroxymelatonin cpd: C05643; N-acetylserotonin cpd: C00978; formyl-5-hydroxykynurenamine cpd: C05647; 4,6-dihydroxyquinoline cpd: C05639; acetyl-CoA cpd: C00024; 2-aminomuconic acid semialdehyde cpd: C03824; 2-aminobenzoic acid cpd: C00108; L-tryptophanyl-tRNA(Trp) cpd: C03512; cinnavalininate cpd: C05640; 4-(2-amino-3-hydroxyphenyl)-2,4-dioxobutanoic acid cpd: C05645; 4-(2-aminophenyl)-2,4-dioxobutanoic acid cpd: C01252; 4,8-dihydroxyquinoline cpd: C05637; indoleacetic acid cpd: C00954; 5-hydroxyindoleacetic acid cpd: C05635; N-methylserotonin cpd: C06212; N-methyltryptamine cpd: C06213
Biosynthesis of unsaturated fatty acids	42	1	0.5983	0.51359	1	1	0	Palmitic acid cpd: C00249	(13Z,16Z)-docosadi-13,16-enoyl-CoA cpd: C16645; tetracosenoyl-CoA cpd: C16532; docosenoyl-CoA cpd: C16531; icosenoyl-CoA cpd: C16530; tetracosanoyl-CoA cpd: C16529; docosanoyl-CoA cpd: C16528; (7Z,10Z,13Z,16Z)-docosatetraenoyl-CoA cpd: C16170; (11Z,14Z)-icosadienoyl-CoA cpd: C16180; (7Z,10Z,13Z,16Z,19Z)-docosapentaenoyl-CoA cpd: C16166; (11Z,14Z,17Z)-icosatrienoyl-CoA cpd: C16179; palmityl-CoA cpd: C00154; stearoyl-CoA cpd: C00412; eicosanoyl-CoA cpd: C02041; oleoyl-CoA cpd: C00510; linoleoyl-CoA cpd: C02050; arachidonyl-CoA cpd: C02249; 8,11,14-eicosatrienoyl-CoA cpd: C03595; gamma-linolenoyl-CoA cpd: C03035; (4Z,7Z,10Z,13Z,16Z,19Z)-docosahexaenoyl-CoA cpd: C16169; (5Z,8Z,11Z,14Z,17Z)-icosapentaenoyl-CoA cpd: C16165; alpha-linolenoyl-CoA cpd: C16162; 13,16-docosadienoic acid cpd: C16533; nervonic acid cpd: C08323; erucic acid cpd: C08316; icosenoic acid cpd: C16526; tetracosanoic acid cpd: C08320; behenic acid cpd: C08281; 7,10,13,16-docosatetraenoic acid cpd: C16527; icosadienoic acid cpd: C16525; clupanodonic acid cpd: C16513; icosatrienoic acid cpd: C16522; palmitic acid cpd: C00249; stearic acid cpd: C01530; arachidic acid cpd: C06425; oleic acid cpd: C00712; linoleic acid cpd: C01595; arachidonic acid cpd: C00219; 8,11,14-eicosatrienoic acid cpd: C03242; gamma-linolenic acid cpd: C06426; (4Z,7Z,10Z,13Z,16Z,19Z)-docosahexaenoic acid cpd: C06429; eicosapentaenoic acid cpd: C06428; alpha-linolenic acid cpd: C06427
Fatty acid biosynthesis	43	1	0.6071	0.49905	1	1	0	Palmitic acid cpd: C00249	Tetradecanoyl-[acp] cpd: C05761; malonyl-[acyl-carrier protein] cpd: C01209; hexadecanoyl-[acp] cpd: C05764; octadecanoyl-[acyl-carrier protein] cpd: C04088; oleoyl-[acyl-carrier protein] cpd: C01203; dodecanoyl-[acyl-carrier protein] cpd: C05223; decanoyl-[acp] cpd: C05755; octanoyl-[acp] cpd: C05752; Hexanoyl-[acp] cpd: C05749; butyryl-[acp] cpd: C05745; trans-hexadec-2-enoyl-[acp] cpd: C05763; (3R)-3-hydroxypalmitoyl-[acyl-carrier protein] cpd: C04633; 3-oxohexadecanoyl-[acp] cpd: C05762; trans-tetradec-2-enoyl-[acp] cpd: C05760; (3R)-3-hydroxytetradecanoyl-[acyl-carrier protein] cpd: C04688; 3-oxotetradecanoyl-[acp] cpd: C05759; trans-dodec-2-enoyl-[acp] cpd: C05758; (R)-3-hydroxydodecanoyl-[acp] cpd: C05757; 3-oxododecanoyl-[acp] cpd: C05756; trans-Dec-2-enoyl-[acp] cpd: C05754; (3R)-3-hydroxydecanoyl-[acyl-carrier protein] cpd: C04619; 3-oxodecanoyl-[acp] cpd: C05753; trans-Oct-2-enoyl-[acp] cpd: C05751; (3R)-3-hydroxyoctanoyl-[acyl-carrier protein] cpd: C04620; 3-oxooctanoyl-[acp] cpd: C05750; trans-Hex-2-enoyl-[acp] cpd: C05748; (R)-3-hydroxyhexanoyl-[acp] cpd: C05747; 3-oxohexanoyl-[acp] cpd: C05746; but-2-enoyl-[acyl-carrier protein] cpd: C04246; (3R)-3-hydroxybutanoyl-[acyl-carrier protein] cpd: C04618; acetyl-[acyl-carrier protein] cpd: C03939; Malonyl-CoA cpd: C00083; acetyl-CoA cpd: C00024; acyl-carrier protein cpd: C00229; Holo-[carboxylase] cpd: C06250; carboxybiotin-carboxyl-carrier protein cpd: C04419; myristic acid cpd: C06424; 3-oxostearoyl-[acp] cpd: C16219; stearic acid cpd: C01530; oleic acid cpd: C00712; dodecanoic acid cpd: C02679; palmitic acid cpd: C00249; acetoacetyl-[acp] cpd: C05744
Primary bile acid biosynthesis	46	1	0.6323	0.45839	1	1	0.02976	Glycine cpd: C00037	Cholesterol cpd: C00187; cholest-5-ene-3beta,26-diol cpd: C15610; 25-hydroxycholesterol cpd: C15519; 7 alpha,26-dihydroxy-4-cholesten-3-one cpd: C17336; 4-cholesten-7alpha,12alpha-diol-3-one cpd: C17339; 7a-hydroxy-cholestene-3-one cpd: C05455; 5-b-cholestane-3a , 7a ,12a-triol cpd: C05454; (25R)-3alpha,7alpha,12alpha-trihydroxy-5beta-cholestan-26-oyl-CoA cpd: C15613; (25S)-3alpha,7alpha,12alpha-trihydroxy-5beta-cholestan-26-oyl-CoA cpd: C17343; 3a,7a,12a-trihydroxy-5b-cholest-24-enoyl-CoA cpd: C05460; 3a,7a,12a-trihydroxy-5b-24-oxocholestanoyl-CoA cpd: C05467; 3a,7a-dihydroxy-5b-24-oxocholestanoyl-CoA cpd: C05449; chenodeoxycholoyl-CoA cpd: C05337; glycine cpd: C00037; taurine cpd: C00245; 3alpha,7alpha,12alpha,26-tetrahydroxy-5beta-cholestane cpd: C05446; 3a,7a,12a-trihydroxy-5b-cholestan-26-al cpd: C01301; 3 beta,7 alpha-dihydroxy-5-cholestenoate cpd: C17335; 7-a,27-dihydroxycholesterol cpd: C06341; 7a-Hydroxycholesterol cpd: C03594; 7-a,25-dihydroxycholesterol cpd: C15520; Choloyl-CoA cpd: C01794; 24-hydroxycholesterol cpd: C13550; 3alpha,7alpha-dihydroxy-5beta-cholestanate cpd: C04554; 3a,7a-dihydroxy-5b-cholestane cpd: C05452; 3 alpha,7 alpha,26-trihydroxy-5beta-cholestane cpd: C05444; 3a,7a-dihydroxy-5b-cholestan-26-al cpd: C05445; (25R)-3alpha,7alpha-dihydroxy-5beta-cholestanoyl-CoA cpd: C17345; (25S)-3alpha,7alpha-dihydroxy-5beta-cholestanoyl-CoA cpd: C17346; 3a,7a-dihydroxy-5b-cholest-24-enoyl-CoA cpd: C05447; 3a,7a,12a-Trihydroxy-5b-cholestanoic acid cpd: C04722; 3a,7a,12a-trihydroxy-5b-cholestanoyl-CoA cpd: C05448; 3a,7a,12a,24-tetrahydroxy-5b-cholestanoyl-CoA cpd: C05450; 3 beta-hydroxy-5-cholestenoate cpd: C17333; (24S)-cholest-5-ene-3beta,7alpha,24-triol cpd: C15518; 7 alpha-hydroxy-3-oxo-4-cholestenoate cpd: C17337; 7a,12a-dihydroxy-5b-cholestan-3-one cpd: C05453; 7a-hydroxy-5b-cholestan-3-one cpd: C05451; chenodeoxycholic acid cpd: C02528; chenodeoxycholic acid glycine conjugate cpd: C05466; taurochenodesoxycholic acid cpd: C05465; 7alpha,25-dihydroxy-4-cholesten-3-one cpd: C17332; cholic acid cpd: C00695; glycocholic acid cpd: C01921; taurocholic acid cpd: C05122; 7 alpha,24-dihydroxy-4-cholesten-3-one cpd: C17331

Purine metabolism	68	1	0.7749	0.25507	1	1	0	Sulfate cpd: C00059	Guanosine diphosphate cpd: C00035; xanthine cpd: C00385; D-ribulose 5-phosphate cpd: C00117; phosphoribosyl pyrophosphate cpd: C00119; L-glutamine cpd: C00064; 5-phosphoribosylamine cpd: C03090; glycineamideribotide cpd: C03838; phosphoribosylformylglycineamidine cpd: C04640; AICAR cpd: C04677; SAICAR cpd: C04823; 5-amino-1-(5-phospho-D-ribosyl)imidazole-4-carboxylate cpd: C04751; RNA cpd: C00046; cyclic AMP cpd: C00575; adenosine triphosphate cpd: C00002; dATP cpd: C00131; ADP cpd: C00008; dADP cpd: C00206; adenosine monophosphate cpd: C00020; adenylsuccinic acid cpd: C03794; inosinic acid cpd: C00130; adenosine cpd: C00212; deoxyadenosine monophosphate cpd: C00360; deoxyadenosine cpd: C00559; deoxyinosine cpd: C05512; xanthosine cpd: C01762; IDP cpd: C00104; guanosine monophosphate cpd: C00144; xanthylic acid cpd: C00655; hypoxanthine cpd: C00262; inosine cpd: C00294; guanine cpd: C00242; deoxyguanosine cpd: C00330; allantoic acid cpd: C00499; uric acid cpd: C00366; 5-hydroxyisourate cpd: C11821; guanosine 3′-diphosphate 5′-triphosphate cpd: C04494; guanosine triphosphate cpd: C00044; 2′-deoxyguanosine 5′-monophosphate cpd: C00362; dGDP cpd: C00361; guanosine cpd: C00387; dGTP cpd: C00286; cyclic GMP cpd: C00942; sulfate cpd: C00059; adenosine phosphosulfate cpd: C00224; phosphoadenosine phosphosulfate cpd: C00053; 5′-Phosphoribosyl-N-formylglycinamide cpd: C04376; inosine triphosphate cpd: C00081; xanthosine 5-triphosphate cpd: C00700; diadenosine tetraphosphate cpd: C01260; P1,P4-bis(5′-xanthosyl) tetraphosphate cpd: C04392; adenosine diphosphate ribose cpd: C00301; adenine cpd: C00147; dIDP cpd: C01344; 2′-deoxyinosine triphosphate cpd: C01345; diadenosine triphosphate cpd: C06197; phosphoribosyl formamidocarboxamide cpd: C04734; 5-hydroxy-2-oxo-4-ureido-2,5-dihydro-1H-imidazole-5-carboxylate cpd: C12248; 5-aminoimidazole ribonucleotide cpd: C03373; DNA cpd: C00039; ammonia cpd: C00014; urea cpd: C00086; (S)-ureidoglycolic acid cpd: C00603; guanosine 3′,5′-bis(diphosphate) cpd: C01228; adenosine 3′,5′-diphosphate cpd: C00054; diguanosine tetraphosphate cpd: C01261; dIMP cpd: C06196; 5-amino-4-imidazolecarboxyamide cpd: C04051; (S) (+)-allantoin cpd: C02350
Aminoacyl-tRNA biosynthesis	69	1	0.7799	0.24863	1	1	0	Glycine cpd: C00037	L-asparagine cpd: C00152; tRNA(Asn) cpd: C01637; L-histidine cpd: C00135; tRNA(His) cpd: C01643; L-phenylalanine cpd: C00079; tRNA(Phe) cpd: C01648; L-arginine cpd: C00062; tRNA(Arg) cpd: C01636; L-glutamine cpd: C00064; tRNA(Gln) cpd: C01640; L-cysteine cpd: C00097; tRNA(Cys) cpd: C01639; glycine cpd: C00037; tRNA(Gly) cpd: C01642; tRNA(Asp) cpd: C01638; L-aspartic acid cpd: C00049; L-serine cpd: C00065; tRNA(Ser) cpd: C01650; L-methionine cpd: C00073; tRNA(Met) cpd: C01647; L-valine cpd: C00183; tRNA(Val) cpd: C01653; L-alanine cpd: C00041; tRNA(Ala) cpd: C01635; L-lysine cpd: C00047; tRNA(Lys) cpd: C01646; L-isoleucine cpd: C00407; tRNA(Ile) cpd: C01644; tRNA(Leu) cpd: C01645; L-leucine cpd: C00123; L-threonine cpd: C00188; tRNA(Thr) cpd: C01651; tRNA(Trp) cpd: C01652; L-tryptophan cpd: C00078; N10-formyl-THF cpd: C00234; L-methionyl-tRNA cpd: C02430; L-tyrosine cpd: C00082; tRNA(Tyr) cpd: C00787; L-proline cpd: C00148; tRNA(Pro) cpd: C01649; L-glutamic acid cpd: C00025; tRNA(Glu) cpd: C01641; glutaminyl-tRNA cpd: C02282; L-asparaginyl-tRNA(Asn) cpd: C03402; tRNA(Sec) cpd: C16636; L-seryl-tRNA(Sec) cpd: C06481; O-phosphoseryl-tRNA(Sec) cpd: C16638; L-histidyl-tRNA(His) cpd: C02988; L-phenylalanyl-tRNA(Phe) cpd: C03511; L-arginyl-tRNA(Arg) cpd: C02163; L-cysteinyl-tRNA(Cys) cpd: C03125; Glycyl-tRNA(Gly) cpd: C02412; L-aspartyl-tRNA(Asp) cpd: C02984; L-seryl-tRNA(Ser) cpd: C02553; L-valyl-tRNA(Val) cpd: C02554; L-alanyl-tRNA cpd: C00886; L-lysyl-tRNA cpd: C01931; L-isoleucyl-tRNA(Ile) cpd: C03127; L-leucyl-tRNA cpd: C02047; L-threonyl-tRNA(Thr)
									cpd: C02992; L-tryptophanyl-tRNA(Trp) cpd: C03512; tetrahydrofolic acid cpd: C00101; N-formylmethionyl-tRNA cpd: C03294; L-tyrosyl-tRNA(Tyr) cpd: C02839; L-prolyl-tRNA(Pro) cpd: C02702; L-glutamyl-tRNA(Glu) cpd: C02987; L-glutamyl-tRNA(Gln) cpd: C06112; L-aspartyl-tRNA(Asn) cpd: C06113; L-selenocysteinyl-tRNA(Sec) cpd: C06482
Pathway	Metabolic pathway name								
Total	The number of metabolites in this pathway								
Hits	The number of differential metabolites hit this pathway								
Raw *p*	*P* value of metabolic pathway enrichment analysis								
−ln (*p*)	Minus log base E of P								
Holm adjust	*P* values corrected by Holm–Bonferroni method for multiple hypothesis testing								
FDR	*P* value corrected by false discovery rate (FDR) method for multiple hypothesis testing								
Impact	Impact value of metabolic pathway topology analysis								
Hits cpd	The names and KEGG IDs of differential metabolites hit the pathway								
Total cpd	All metabolite names and KEGG IDs contained in this pathway								

## Data Availability

All data generated or analyzed during this study are included in this article.
